# Person Features and Lexical Restrictions in Italian Clefts

**DOI:** 10.3389/fpsyg.2019.02105

**Published:** 2019-09-20

**Authors:** Cristiano Chesi, Paolo Canal

**Affiliations:** Research Center for Neurocognition, Epistemology and Theoretical Syntax (NETS), School of Advanced Studies, Istituto Universitario di Studi Superiori (IUSS), Pavia, Italy

**Keywords:** pronominal determiners, *top-down* derivation, complexity, cue-based retrieval, object cleft, intervention, similarity, memory load

## Abstract

In this paper, we discuss the results of two experiments, one off-line (acceptability judgment) and the other on-line (eye-tracking), targeting Object Cleft (OC) constructions. In both experiments, we used the same materials presenting a manipulation on person features: second person plural pronouns and plural definite determiners alternate in introducing a full NP (“it was [_DP1_ the/you [_NP_ bankers]]_i_ that [_DP2_ the/you [_NP_ lawyers]] have avoided __i_ at the party”) in a language, Italian, with overt person (and number) subject-verb agreement. As results, we first observed that the advantage of the bare pronominal forms reported in previous experiments (Gordon et al., 2001; Warren and Gibson, 2005, a.o.) is lost when the full NP (the “lexical restriction” in Belletti and Rizzi, 2013) is present. Second, an advantage for the mismatch condition, *Art*_1_*-Pro*_2_, in which the focalized subject is introduced by the determiner and the OC subject by the pronoun, as opposed to the matching *Pro*_1_*-Pro*_2_ condition, is observed, both off-line (higher acceptability and accuracy in answering comprehension questions after eyetracking) and on-line (e.g., smaller number of regressions from the subject region); third, we found a relevant difference between acceptability and accuracy in comprehension questions: despite similar numerical patterns in both off-line measures, the difference across conditions in accuracy is mostly not significant, while it is significant in acceptability. Moreover, while the matching condition *Pro*_1_*-Pro*_2_ is perceived as nearly ungrammatical (far below the mean acceptability across-conditions), the accuracy in comprehension is still high (close to 80%). To account for these facts, we compare different formal competence and processing models that predict difficulties in OC constructions: similarity-based (Gordon et al., 2001, a.o.), memory load (Gibson, 1998), and intervention-based (Friedmann et al., 2009) accounts are compared to processing oriented ACT-R-based predictions (Lewis and Vasishth, 2005) and to *top-down Minimalist* derivations (Chesi, 2015). We conclude that most of these approaches fail in making predictions able to reconcile the competence and the performance perspective in a coherent way to the exception of the *top-down* model that is able to predict correctly both the on-line and the off-line main effects obtained.

## Introduction

A necessary condition for comprehending correctly an Object Relative clause (OR) is to interpret the head of this construction [“the banker” in (1)] as the direct object of the predicate within the relative clause (“praised”):


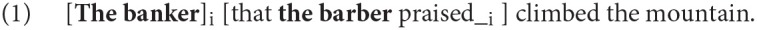


The fact that ORs are generally harder to process than Subject Relative (SR) clauses^1^ has been shown systematically using self-paced reading experiments (since King and Just, 1991, a.o.), probe-task paradigms (Wanner and Maratsos, 1978, a.o.), eye movements tracking (Traxler et al., 2002, a.o.), or by monitoring the electrical (Weckerly and Kutas, 1999) or metabolic (Just et al., 1996, a.o.) activity of the brain. Focusing on ORs, Bever (1974) first noticed that their difficulty can be mitigated by varying the type of subject within the relative clause (examples from Gordon et al., 2001,^2^):


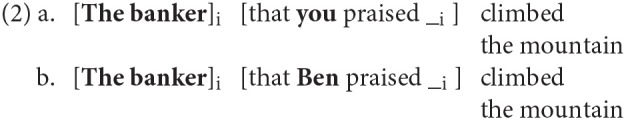


When pronouns are processed in the subject position within the relative clause, as in (2).a, self-paced reading experiments show that the critical verbal regions (“praised” and “climbed”) are read faster than when proper names are present, (2).b; when definite descriptions occupy both the head and the subject position, we obtain the slowest performance on the same critical verbal regions, as in (1)^3^.

This effect has been extensively studied both from the theoretical/competence perspective (Friedmann et al., 2009; Belletti and Rizzi, 2013, a.o.) and from the psycholinguistic/performance one (Gordon et al., 2004, a.o.), especially in Object Clefts (OCs), when both the subject and the focalized DP can be definite descriptions, proper names or pronouns, (3) (Warren and Gibson, 2005):


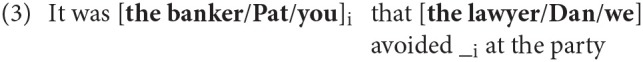


In this paper, we present a manipulation of person features [3rd (default) vs. 2nd person] in the paradigm in (3) to investigate the role of person agreement in Italian (an overt subject-verb person agreement language) under the presence of a “lexical restriction” (i.e., the NP introduced by the determiner): second person pronouns will be used as determiners and compared to definite articles under the presence of a plural lexical restriction, as exemplified in (4):


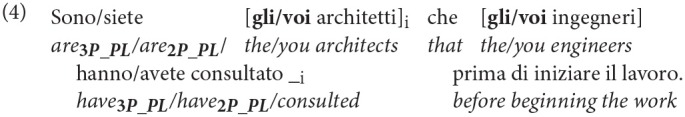


This study consists of two new experiments (section Materials and Methods): an acceptability judgment (experiment 1) and an eyetracking study (experiment 2). Comparing off-line (acceptability scores in experiment 1 and accuracy in answering comprehension questions after eyetracking in experiment 2) and on-line evidence (all relevant eyetracking measures in experiment 2) we will evaluate the actual fit of some prominent model discussed in literature (section Predicting Processing Difficulties) aiming at explaining the contrasts revealed in (3): from the analysis (section Discussion) of the results of our experiments (section Results) we conclude that none of the models presented readily predicts the behavioral evidence revealed by this study. We will then argue in favor of the left-right, *top-down* derivational minimalist perspective (Chesi, 2015) where the “complexity” of the non-local dependency is computed using a Feature Retrieval and Encoding Cost (FREC) function: this model better integrates the on-line and off-line results gathered here.

In the first part of this paper, we will introduce the structural properties of the Object Cleft sentences under analysis (section The Properties of the Object Clefts (OCs) Under Analysis). A brief state-of-the-art summary on OC processing effects [section Processing Object Clefts (OCs)] will precede the summary of five major models and their predictions on the contrast previously tested: similarity-based (Gordon et al., 2001, section Similarity-Based Accounts), intervention-based (Friedmann et al., 2009, section Intervention-Based Accounts), and Dependency Locality Theory (DLT, Gibson, 1998, 2000, section *memory-load* Accounts) models will be compared with processing-oriented, memory-usage models that make explicit predictions on reading times and are possibly more transparent in terms of brain mechanisms involved: Lewis and Vasishth (2005) model based on ACT-R (section ACT-R-Based Predictions) and a *top-down*, left-right minimalist derivation based on Chesi (2015) (section *top-down* (Left-Right) Minimalist Derivations). Then we will concentrate on person features on the DP triggering overt verbal agreement, especially focusing on pronouns used as determiners (section Pronouns as Determiners and Agreement): this should clarify the rationale behind the proposed manipulation and the semantic/pragmatic impact of this construction on processing.

## The Properties of the Object Clefts (OCs) Under Analysis

Object Clefts (OCs) are peculiar focalization constructions in which a direct object is displaced in a prominent left-peripheral position (Rizzi, 1997). Following Belletti's (2008) analysis, in these structures the copula selects a truncated CP in which the object was moved into the FocP position as shown below:


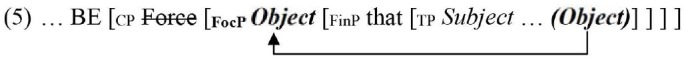


According to Belletti, OCs, as opposed to Subject Clefts^4^ (SCs), can only convey contrastive/corrective focus (this is the role of FocP), then its realization will be felicitous and perfectly natural only in specific contexts. For instance in correcting/rectifying a statement as below:

**Table:** 

(6) X:	Ho sentito che Alberto ha salutato qualcuno prima di partire per le vacanze; ha per caso salutato Beatrice prima di partire? (Dopo il litigio che hanno avuto per colpa di Claudia sarebbe stato un segno distensivo) *I heard that A. said goodbye to someone before leaving for holidays; has he said good bye to B. before leaving? (After the fight they had because of C., this would have been a positive sign)*
Y:	(no, non-era Beatrice, purtroppo) era CLAUDIA che Alberto ha salutato prima di partire! *(no, it wasn't B, unfortunately) it was C._focalized_ that A. said goodbye to before leaving!*

Notice that a presupposition of existence [p.c. Benincà in (Belletti, 2008), (7).a] and uniqueness, as well as exhaustivity [as in Identificational Focus discussed in E. (Kiss, 1998), (8)], are also implied by the cleft constructions [contra standard focalization, both in root, (7).b, or in embedded contexts, (7).c]:

(7) a. ^*^(non) è NESSUNO che ho incontrato (non-tutti)
  it is (not) NOBODY that I met (not everybody)
b. NESSUNO ho incontrato (non-tutti)
  NOBODY I met (not everybody)
c. ho detto che NESSUNO assumeranno (non-tutti)
  I have said that NOBODY they will hire (not everybody)

(8) è UNA MELA che ho mangiato (non-una pera o qualcos'altro)
it is AN APPLE that I have eaten (not a pear or anything else)

Despite their peculiarities, these are perfect configurations for testing non-local crossing dependencies in comprehension: from a processing perspective, the distal argument (the focalized DP) must be retained in memory and retrieved, later on, when the verbal predicate is encountered, crucially after the subject has been interpreted as the agent of the predication. Notice that the absence of an appropriate context does not preclude the possibility of correctly processing and interpreting these constructions: in all the experiments that will be mentioned in the next section [section Processing Object Clefts (OCs)], any context introducing OCs was absent.

### Processing Object Clefts (OCs)

The performance contrasts elicited by OCs suggest that the nature of both DPs present in the construction plays a major role (Gordon et al., 2001, 2004; Warren and Gibson, 2005): a definite DP (D), a proper name (N), or a pronoun (P) occupying the two relevant positions produce different effects according to their relative distribution. The full prototypical paradigm (Warren and Gibson, 2005), introduced in (3) and expanded below in (9) for convenience, is used to illustrate these contrasts.

**Table:** 

(9)		*object_*focalized*_*	*subject*	*verb*	*spill-over*	*condition*
	a.	It was **the banker**	that **the lawyer**	**avoided _**	at the party	*[D_1_-D_2_]*
	a'.	It was **the banker**	that **Dan**	**avoided _**	at the party	*[D_1_-N_2_]*
	a”.	It was **the banker**	that **we**	**avoided _**	at the party	*[D_1_-P_2_]*
	b.	It was **Patricia**	that **the lawyer**	**avoided _**	at the party	*[N_1_-D_2_]*
	b'.	It was **Patricia**	that **Dan**	**avoided _**	at the party	*[N_1_-N_2_]*
	b”.	It was **Patricia**	that **we**	**avoided _**	at the party	*[N_1_-P_2_]*
	c.	It was **you**	that **the lawyer**	**avoided _**	at the party	*[P_1_-D_2_]*
	c'.	It was **you**	that **Dan**	**avoided _**	at the party	*[P_1_-N_2_]*
	c”.	It was **you**	that **we**	**avoided _**	at the party	*[P_1_-P_2_]*

Warren and Gibson (2005) evidence [average reading times reported in (10)^5^], based on the paradigm in (9), shows that the greatest slowdown in self-paced reading at the critical verbal segment (*avoided*) is associated to the *D*_2_ condition [i.e., when the subject of the cleft is a definite description, (9).a,b,c]. This correlates with the lowest accuracy rate in comprehension questions. Similar (non-significantly different) reading times are revealed for the *N*_1_*-N*_2_ matching condition (9).b′, while the *P*_1_*-P*_2_ matching condition (always presenting a person mismatch), as well as the other conditions in which *P* is the subject of the cleft, produce the fastest reading times of the critical verbal region.

**Table:** 

(10)	**Condition**	**Average RT** **(SE) ms**
	**D**_**1**_**-D**_**2**_	365 (19)
	**D**_**1**_**-N**_**2**_	319 (12)
	**D**_**1**_**-P**_**2**_	306 (14)
	**N**_**1**_**-D**_**2**_	348 (18)
	**N**_**1**_**-N**_**2**_	347 (21)
	**N**_**1**_**-P**_**2**_	291 (14)
	**P**_**1**_**-D**_**2**_	348 (18)
	**P**_**1**_**-N**_**2**_	311 (15)
	**P**_**1**_**-P**_**2**_	291 (13)

The authors reported a reliable effect of subject type with *P*_2_ conditions averaging 30 ms faster than *N*_2_ conditions, which is 28 ms on average faster than *D*_2_ conditions. A marginally significant interaction (mainly driven by the slowest *D*_1_*-D*_2_ and *N*_1_*-N*_2_ conditions) indicates that the matching conditions, overall, are significantly slower than the mismatch conditions.

Also in comprehension, Warren and Gibson reported a main effect on the subject type (*D*_2_ condition is harder than *N*_2_, which is harder than *P*_2_) and an interaction between focalized object type and subject type, with all matching conditions inducing lower accurate results.

These results confirmed and expanded other results discussed in literature (e.g., Gordon et al., 2001).

### Predicting Processing Difficulties

Various models have been proposed to account for the performance data presented in the previous section. Here five models will be discussed, all considering as key factors: (i) the nature of the DPs involved in the dependency (*memory-load* account, Warren and Gibson, 2005, section *memory-load* Accounts), (ii) the similarity between the two DPs (*similarity-based*, Gordon et al., 2001 and section Similarity-Based Accounts *intervention-based* Friedmann et al., 2009, section Intervention-Based Accounts, accounts), (iii) the distance/activation of the focalized object with respect to the predicate (Lewis and Vasishth, 2005, section ACT-R-Based Predictions), or a combination of these factors (*top-down Minimalist* account, Chesi, 2015 section *top-down* (Left-Right) Minimalist Derivations). The predictions these models differ substantially both in terms of general complexity factor (DP types vs. matching/mismatching conditions), relevant features inducing difficulty and the exact moments in which such difficulty can be revealed (encoding at DP vs. retrieval at VP; on-line as slow down at specific regions vs. off-line as comprehension accuracy).

#### Similarity-Based Accounts

Gordon et al. (2001) explicitly focus on working memory demands in their studies using self-paced reading paradigms. Their proposal is based on the idea that having two DPs “of the same kind” stored in memory makes the OR/OC more complex than SR/SC. This is sufficient to model memory interference during encoding, storage, and retrieval (Crowder, 1976). When similarity between DPs is calculated considering noun type (proper vs. common), gender, number, animacy, case, and person, this theory is sufficient to predict asymmetries for most of the contrasts presented in section Processing Object Clefts (OCs): *D*_1_*-D*_2_ and *N*_1_*-N*_2_ matching condition are expected to be the hardest configurations, while the *P*_1_*-P*_2_ matching configuration might result slightly easier than the other matching conditions because of person features mismatch. In all other mismatch cases, this approach predicts (both on-line and off-line) lighter effects because of the difference in type/features without being able to distinguish between *D*_1_*-N*_2_ [easier, according to (10)] and *N*_1_*-D*_2_ [harder in (10)] conditions or between *P*_1_*-D*_2_ [harder in (10)] and *D*_1_*-P*_2_ [easier in (10)]. This is also expected under the assumption that all features equally contribute to memory confusion.

#### Memory-Load Accounts

*Memory-load* accounts (Gibson, 1998; Warren and Gibson, 2002, 2005, a.o.) explain most of the contrasts presented in section Processing Object Clefts (OCs) by postulating an “integration cost” (Gibson, 1998, Syntactic Prediction Locality Theory, SPLT) proportional to new discourse referents^6^: since pronouns do not introduce new discourse referents and names are referentially lighter than definite descriptions (Warren and Gibson, 2005), memory-load accounts predict faster reading times at the cleft verbal region when the subject is a pronoun and slightly longer reading times when it is a proper name. On the other hand, this account incorrectly predicts faster reading times for the *N*_1_*-N*_2_ matching condition (“it was Patricia that Dan *avoided* at the party”) than for the *D*_1_*-D*_2_ condition (“it was the lawyer that the businessman *avoided* at the party”), even though no significant differences emerged from this contrast.

#### Intervention-Based Accounts

The intervention-based accounts (Friedmann et al., 2009; Belletti and Rizzi, 2013, a.o.) can explain the symmetry revealed in the *D*_1_*-D*_2_ and *N*_1_*-N*_2_ matching conditions in terms of similarity of the critical intervening features: Friedmann et al. (2009), building on Rizzi (1990) locality constraint, assume that whenever features are shared between a filler, *X* [e.g., “the banker” in (1)] and a structural intervener, *Z* [e.g., “the barber” in (1)], the relation between *X* and the related selected gap, *Y*, gets disrupted in a way that is proportional to the kind (and number) of features involved. Assuming that lexical restriction, rather than referentiality (c.f. section *memory-load* Accounts), is computed and that features expressing such lexical restriction in definite descriptions, proper names and pronouns are distinct (they assume *N* for common nouns, *N*_*prop*_ for proper names, and a null *N* for pronouns), the intervention-based account predicts exactly that the matching conditions in which common nouns and proper names are present are comparable, while pronouns induce easier processing since *N* is absent. A crucial assumption here is that only features triggering movement should cause intervention (Friedmann et al., 2009:83). In this respect, the lexical restriction should not play a significant role, since this “feature” is buried within the DP and does not seem to trigger movement. However, (Belletti and Rizzi, 2013) explicitly consider the lexical restriction as a movement trigger^7^, hence rescuing the idea that its presence has an impact in terms of intervention. This model does not predict differences when pronouns are in the focalized object or in the subject position, neither it makes explicit predictions in the *D*_1_*-N*_2_ and *N*_1_*-D*_2_ cases: being *N* and *N*_*prop*_ distinct, either we assume that they play a role in triggering movement, hence expecting milder effects than in the matching conditions, or we assume that *N* and *N*_*prop*_ are not involved in movement hence these cases should be comparable with respect to the other matching cases. Since verb region in the *D*_1_*-N*_2_ is significantly faster than in the *N*_1_*-D*_2_ condition according to (10), neither assumptions lead to the correct prediction.

#### ACT-R-Based Predictions

Lewis and Vasishth (2005) present an explicit moment-by-moment model of parsing^8^ based on independently motivated working memory principles. Their predictions, both on similarity effects (c.f. section Similarity-Based Accounts) and probability to retrieve the correct, accessible, syntactic chunk over time (c.f. section *memory-load* Accounts), follow from their assumptions based on an implementation of some components of the Adaptive Control of Thought-Rational (ACT-R) architecture (Anderson and Matessa, 1997; Anderson, 2005) within the sentence comprehension perspective. By focusing on working-memory retrieval, this model is able to estimate precisely the integration cost of a non-local constituent relying on its distance from its re-attachment point: the structural chunks are stored in memory and their activation (i.e., a purely numerical value) fades over time; stored chunks receive an activation boost whenever re-accessed. The longer is the time passed after the last re-activation, the longer it will take to retrieve the correct chunk. This plainly explains the difference between retrieving the subject in a SC, which is relatively fast, or the focalized object in OCs, which is relatively slow due to the time spent in attaching the intervening subject. This model can be used to simulate memory decay and difficulty in re-accessing specific constituents. Moreover, since their attempt is to explicitly describe processes and memory structures giving rise to specific linguistic configuration by providing a psychologically motivated theory of processing, this approach has a high explanatory potential. However, unless specific cues pre-activate the object (e.g., agreement as in clitic doubling constructions), this model can hardly predict the relevant asymmetries in the paradigm discussed in (9): we could assume retrieval and attachment of 1st and 2nd person pronouns to be slightly faster than default 3rd person DPs because of their higher saliency in the context; in this sense when P is in the focalized position (*P*_1_) or in the subject position (*P*_2_), this might somehow reduce the general cost paid for retrieving a distal argument, but any prediction in all other cases requires extra assumptions.

#### Top-Down (Left-Right) Minimalist Derivations

An alternative way to look at processing, without assuming any specific parsing algorithm or declarative grammatical rule format (as in Lewis and Vasishth, 2005), while maintaining an incremental left-right perspective, is presented in Chesi (2015, 2017): in the *Minimalist top-down* derivation proposed, *merge* is the sole structure building operation and it operates by attaching new (incoming) items always to the right of the phrase structure built so far (c.f. Phillips', 1996, *merge right*). The integration of new items is guided by the expectations triggered by the select feature(s) lexically encoded in the items already merged: for instance, a lexical entry like [_V_ run _= D_]^9^ indicates that the verb (“V”) “run” selects a determiner (“=D”), namely that a DP is expected next; expectations are always projected after the lexical item is processed. If a category [_X_ ] is expected (as result of the expansion of “=X” select feature) either a new [_X_ *item*] or [_X__Y_ *item*] can be merged next; in the second case, [_Y_ <*item*>] must be stored in memory since the “Y” categorial feature was unexpected (i.e., unselected) and must be remerged in the structure as soon as a lexical item with a “=Y” select feature is processed. This is how a non-local, filler-gap dependency (movement) is implemented. In (11), we exemplify the OC derivation using the grammatical knowledge (*Lex*) for the relevant paradigm in (9)^10^:


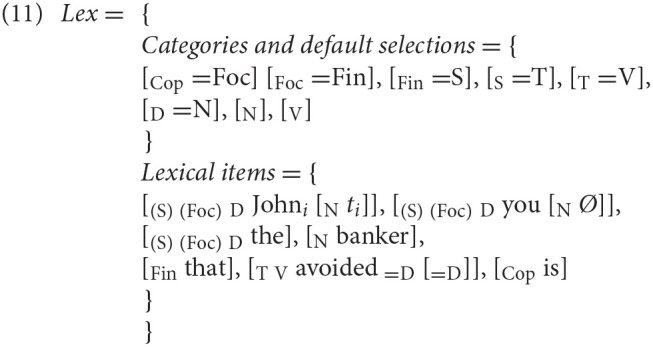


The relevant part of the derivation (equivalent both in generation and in parsing) can be schematized as follows:

(12) (It) is the John that you avoided …


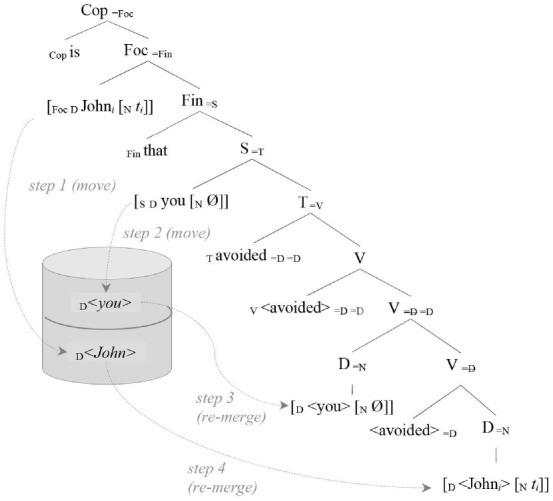


This tree diagram summarizes the history of the derivation, which implements the OC analysis presented in (5) and it is transparent with respect to processing (both parsing and generation).

As we see after step 1 and step 2, both arguments must be stored in memory because of their unexpected (i.e., unselected) “argumental” (D) feature right after they get first merged into the structure. Both arguments are retrieved after the verb is merged and its selection requirements are expressed (steps 3 and 4). To predict processing difficulties at retrieval, we associate a cost to the memory buffer access: this cost grows exponentially with respect to the number of items stored (*m*), linearly with respect to the number of new features to be retrieved from memory (*nF*), and it is mitigated (linearly, again) by the number of distinct cued features (*dF*) by *x* (the region where retrieval is requested, in this case the verbal predicate). This is the core of the Feature Retrieval Cost (*FRC*)^11^ function:

(13) $FRC(x)\;=\overset{n}{\underset{i=1}{\prod }}\frac{{\left(1+nFi\right)}^{m_{i}}}{\left(1+dFi\right)}$

Using the lexicon in (11), we expect to retrieve definite descriptions like [_D_ *the* [_N_ *lawyer*]] (namely a *D* and a *N* category), proper names like *John* = [_D_ *John*_*i*_ [_N_ *t*_*i*_]] (i.e., a contextually salient *D* index and a coindexed *N*) and pronouns like *you* = [_D_ *you* [_N_ Ø ]] (i.e., just a contextually salient *D* index; for more details on these DP analyses, see section Pronouns as Determiners and Agreement). With these feature structures, we obtain the following FRCs at the verb segment for each conditions in (9). These can be easily compared with the average reading times in (10):

**Table:** 

(14)	**Condition**	**Average RT (SE) ms**	**log(FRC)^12^**
	**D**_**1**_**-D**_**2**_	365 (19)	1,43
	**D**_**1**_**-N**_**2**_	319 (12)	1,08
	**D**_**1**_**-P**_**2**_	306 (14)	0,78
	**N**_**1**_**-D**_**2**_	348 (18)	1,26
	**N**_**1**_**-N**_**2**_	347 (21)	1,26
	**N**_**1**_**-P**_**2**_	291 (14)	0,6
	**P**_**1**_**-D**_**2**_	348 (18)	1,26
	**P**_**1**_**-N**_**2**_	311 (15)	0,9
	**P**_**1**_**-P**_**2**_	291 (13)	0,6

Pearson correlation between average reading times and log(FRC) is extremely significant: *r*(7) = 0.98, *p* < 0.001.

Notice that the FRC plainly subsumes (and integrates) Friedmann et al. (2009) account. Moreover, it precisely characterizes the triggers of the filler-gap dependency similarly to the cue-based memory retrieval approach (Van Dyke and McElree, 2006): we expect confusion (higher FRC) when the cued characteristic features are non-unique at retrieval. This also explains the cross-linguistic variation revealed, for instance, in Hebrew vs. Italian with respect to gender vs. number (Belletti et al., 2012): since in Hebrew the verb agrees with its subject also in gender, in Hebrew, but not in Italian, gender mismatch facilitates ORs processing. Under this perspective, this is because the verb uses such cues to retrieve the relevant argument from memory (hence, in FRC terms, gender mismatch increases *dF* in Hebrew since cued by the verb), while just number mismatch, but not gender mismatch, helps in Italian for the same reason (*dF* increases when cued number is in a mismatch configuration).

In addition to FRC, an encoding cost must be considered whenever an element is merged into the structure (similarly to Gibson's 1998 new discourse referent cost): this is the Feature Encoding Cost (*FEC*), a numerical value associated to each new item merged that is proportional to the number of new relevant features integrated in the structure:

(15) $FEC(x)=\overset{n}{\underset{i=1}{\sum }}eF_{i}$

*eF* is the cost of each new relevant feature to be encoded at *x*. For simplicity *eF* = 1 for a new categorial feature introduced (e.g., 1 for D and 1 for N), 2 for a duplication of the same lexical category still requiring a structural integration as selected argument (i.e., 2 for the second N both in *D*_1_*-D*_2_ and *N*_1_*-N*_2_), 0 otherwise. In the paradigm (9) the FEC predictions are the following ones:

**Table:** 

(16)			*object*_*focalized*_		*subject*	*verb*	*spill-over*	*condition*
	a.	It was	**the banker**	that	**the lawyer**	**avoided _**	at the party	[D_1_-D_2_]
		1	2	1	3	2	3	
	a'.	It was	**the banker**	that	**Dan**	**avoided _**	at the party	[D_1_-N_2_]
		1	2	1	1	2	3	
	a”.	It was	**the banker**	that	**we**	**avoided _**	at the party	[D_1_-P_2_]
		1	2	1	0	2	3	
	b.	It was	**Patricia**	that	**the lawyer**	**avoided _**	at the party	[N_1_-D_2_]
		1	1	1	2	2	3	
	b'.	It was	**Patricia**	that	**Dan**	**avoided _**	at the party	[N_1_-N_2_]
		1	1	1	2	2	3	
	b”.	It was	**Patricia**	that	**we**	**avoided _**	at the party	[N_1_-P_2_]
		1	1	1	0	2	3	
	c.	It was	**you**	that	**the lawyer**	**avoided _**	at the party	[P_1_-D_2_]
		1	0	1	2	2	3	
	c'.	It was	**you**	that	**Dan**	**avoided _**	at the party	[P_1_-N_2_]
		1	0	1	1	2	3	
	c”.	It was	**you**	that	**we**	**avoided _**	at the party	[P_1_-P_2_]
		1	0	1	0	2	3	

More precisely, in accordance with the structural assumptions expressed in (11), definite descriptions generally require the encoding of two critical new features (a determiner and a nominal restriction), proper names one (the contextually salient determiner is “free” and the proper name nominal restriction costs 1), while deictic pronouns have no encoding cost since contextually already present in the context (hence already pre-activated).

The absence of cost and the extra cost associated, respectively, to an already introduced feature and to the duplication of a category is coherent with a conception of memory as a pattern associator: if the pattern *p*_*f*_, encoding feature *f*, has been just activated, re-activating it should have a minor cost (priming effect), while forcing a differentiation in a fully-overlapping pattern should induce the recruitment of extra memory units, hence an extra cost.

#### Summary

Summarizing, Table 1 reports the predictions made by the models just described and the average reading times revealed at the verb segment in (9) [data from (10)]:

**Table 1:** Summary of the predictions for the paradigm in 10 (data from Warren and Gibson, 2005).

**Condition**	**D_**1**_-D_**2**_**	**D_**1**_-N_**2**_**	**D_**1**_-P_**2**_**	**N_**1**_-D_**2**_**	**N_**1**_-N_**2**_**	**N_**1**_-P_**2**_**	**P_**1**_-D_**2**_**	**P_**1**_-N_**2**_**	**P_**1**_-P_**2**_**
**Read. time**	**365**	**319**	**306**	**348**	**347**	**291**	**348**	**311**	**291**
**(SE) ms**	**(19)**	**(12)**	**(14)**	**(18)**	**(21)**	**(14)**	**(18)**	**(15)**	**(13)**
Memory-load prediction	Hard	Medium	Easy	Hard	Medium	Easy	Hard	Medium	Easy
Similarity-based prediction	Hard	Medium	Easy	Medium	Hard	Easy	Easy	Easy	Medium
Intervention-based prediction	Hard	?	Easy	?	Hard	Easy	Easy	Easy	Easy
ACT-R-based prediction	Hard	Hard	Medium	Hard	Hard	Medium	Medium	Medium	Easy
Top-down prediction	Hardest	Medium	Medium–Easy	Hard	Hard	Easy	Hard	Medium	Easy

Theories based on the referentiality hierarchy (Ariel, 1990; Gibson, 1998; Warren and Gibson, 2005, a.o.; *memory-load* prediction in the table) fail to predict that also *N*_1_*-N*_2_ matching condition induces a low performance comparable to the *D*_1_*-D*_2_ matching condition. Similarity-based accounts (Gordon et al., 2004, a.o.) capture this fact, but fail in distinguishing any order permutation in mismatching conditions (e.g., *D*_1_*-P*_2_ vs. *P*_1_*-D*_2_); Intervention-based accounts (e.g., Belletti and Rizzi, 2013, a.o.) correctly predict harder times with both *D*_1_*-D*_2_ and *N*_1_*-N*_2_ matching condition, also expecting better performances with pro-intervening conditions (i.e., *D*_1_*-P*_2_, *N*_1_*-P*_2_, *P*_1_*-P*_2_), but fail in predicting any distinction among other conditions (e.g., *P*_1_*-D*_2_ vs. *D*_1_*-P*_2_ or *D*_1_*-N*_2_ vs. *D*_1_*-N*_2_). Notice moreover that the processing costs at the verb segment do not follow from this perspective (as in any other bottom-to-top, movement-based approach). The ACT-R-based models, only relying on distance and pre-activation of the relevant argument, can predict easier retrieval only when *P* is present either at the subject or at the focalized object position. Also in these cases, *P*_1_*-D*_2_ (and more marginally *P*_1_*-N*_2_) would be predicted to be easier than it actually is. In the end, the *top-down* model (Chesi, 2015) correctly predicts more efforts in processing the *D*_1_*-D*_2_ and *N*_1_*-N*_2_ matching conditions (with *D*_1_*-D*_2_ being the hardest configuration), medium difficulty when *D*_2_ and *N*_2_ are integrated in the subject position (different encoding costs) and lighter effects when *P*_2_ is present because of case (nominative “we” vs. “us” morphology in English, while 1st/2nd vs. 3rd person asymmetry can not be used as a cue because of past tense of the cleft predicate).

In conclusion, memory-load and *top-down* predictions present the highest level of correlation with respect to the revealed average reading times, but they make quite different predictions: in *memory-load* theories interference is irrelevant, while the *top-down* prediction crucially relies on the fact that retrieval is at issue, especially when features overlapping among items to be re-merged occurs. For the *top-down* model, also an encoding cost is considered, but the prediction is that the items already present (salient) in the discourse environment and, more generally, those features already merged in the structure, pay a minor cost at encoding (providing a precise characterization of the “more accessible” referents in memory-load accounts), with the exception of the re-introduction of a categorial feature (*N* in this case) and, possibly, its saliency specification (D), whenever they must be kept distinct in memory.

### Pronouns as Determiners and Agreement

One way to dig further into the predictions emerging from these different assumptions is to keep all peculiar factors of OCs constant while investigating the specific contribution of single cued features using an overt subject-verb agreement language: focusing on person features, 1st and 2nd person, unlike 3rd person, are anchored to the speech event, being always present in a speech act (and in a left-peripheral structural dedicated position, Bianchi, 2003, 2006; Sigurdhsson, 2004). Because of their saliency (and dedicated structural position), we might expect 1st and 2nd person features to facilitate the integration of an argument better than default 3rd person (a non-person, in Sigurdhsson, 2004 terms). This could have an impact in terms of encoding: “highly accessible” deictic pronouns are lighter both for the *memory-load* (higher position in the accessibility hierarchy, section *memory-load* Accounts) and for the *top-down* models (being already present in phrase structure, they do not pay an extra FEC, section *top-down* (Left-Right) Minimalist Derivations).

On the other hand, at retrieval, different hypotheses can be formulated: considering person mismatch as a general facilitation (default hypothesis, H1), both the *similarity-based* (under any condition) and the *top-down* model (only when the relevant person mismatch is cued by the verbal agreement morphology) predict a facilitation^13^; an alternative hypothesis (H2), considering the salience of 1st and 2nd person features, should predict a facilitation only for 1st and 2nd person and not for 3rd person. Under this second hypothesis, the only model making different predictions, based on the arguments involved, is the *top-down* model: only the subject and only when verb agreement is overt, 1st/2nd vs. 3rd person mismatch should produce a facilitation [this is because only 1st/2nd person feature mismatch would be considered as a *dF* facilitation for the FRC in (13)]. A neurophysiological evidence supporting the idea that (1st/2nd vs. 3rd) person features are peculiar in terms of subject-verb agreement (vs. number) is discussed in Mancini et al. (2011).

Notice that the introduction of a lexical restriction after the pronoun (e.g., “[_D_ you [_N_ bankers]]”) would remove any advantage of the bare pronominal condition according to the Intervention-based model (section Intervention-Based Accounts) and, for different reasons (increased number of features to be retrieved and compared), for the *top-down Minimalist* model (section *top-down* (Left-Right) Minimalist Derivations), but not for the other models. According to Belletti and Rizzi (2013) pronouns are to a lesser extent interveners because of their lack of a lexical restriction. In fact, pronouns, given an appropriate context, can function as determiners (Postal, 1966, a.o.) and, unlike determiners, bear person features other than default 3rd person.

On the usage of pronouns as determiners^14^, we refer to Elbourne (2005) analysis and we consider them as (empty) definite determiners taking an index and an NP predicate as arguments. According to Elbourne (2005), this is the structure shared by all DPs referring to individuals, namely proper names, pronouns and definite descriptions. In this sense, the “lexical restriction” would be an NP predicate which is, semantically speaking, denoted by type <*e,t*>, while the denotation of the pronominal determiner (“you”) is expressed as follows^15^:

(17) $\begin{array}{c} {\left[\left[you_{i}\right]\right]}^{g,a}=\;\lambda f\;:\;f\;\in \;D_{<e,t>\;}\;\&\;a\;\leq_{i}\\ g\left(j\right)\;\&\;f\left(g\left(j\right)\right)=1.g\left(j\right) \end{array}$

This means that “you,” when used as a determiner in the construction “you bankers,” takes the NP [“banker(s)”] with denotation *f* and returns, as the denotation of the full DP, some contextually salient plural individual *j*, such that the addressees *a* (deictic use of “you”) must be part of *j* and *j* must be *f* (i.e., a banker).

The contextual salience of the relevant individuals is necessary for the sentence to be acceptable and it must be postulated in out of the blue sentences; this means that if a relevant context is not provided to the reader, s/he must infer by her/himself that a salient group of individuals is presupposed by the sentence even if s/he does not share this information with the speaker at the utterance time. On the one hand, we might expect this missing contextual information, related to unexpected saliency, to produce some slowdown in processing, forcing the reader to update his knowledge of the common ground in order to accept this specific utterance; on the other, this is a perfectly grammatical construction and it should be correctly interpreted even when an appropriate context is missing. As far as we can tell, the presence/absence of an appropriate context licensing this usage of second person pronouns as determiners has never been tested before.

Considering the cleft sentences under analysis, this could even happen twice:


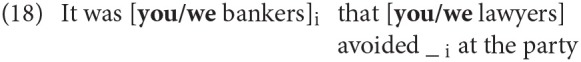


Both the focalized DP and the cleft subject require that both a group of bankers and a group of lawyers be salient in the context and that the speaker and/or the addressee be part of one specific group. If the context is provided and the two groups of bankers and lawyers are in the common ground, the sentence should sound perfectly acceptable, if not, the reader should postulate the presence of the two groups after she/he realizes that none of them was accessible in her/his contextual knowledge. To our knowledge, this again has never been tested before.

Assuming a given cost for definite descriptions, a pronominally restricted DP (“you bankers”) would pay either the same cost (default assumption), a minor cost as pronouns (coherently with their implicit referentiality in the *memory-load* model) or an extra cost (whenever they are non-salient in the context or they get re-introduced twice, as predicted in the *top-down* model).

### Testing the Different Predictions

The paradigm expanded in (19) will be used to test the specific contribution of 2nd vs. 3rd person (default) in an overt subject verb agreement language, Italian, under the assumptions previously discussed:


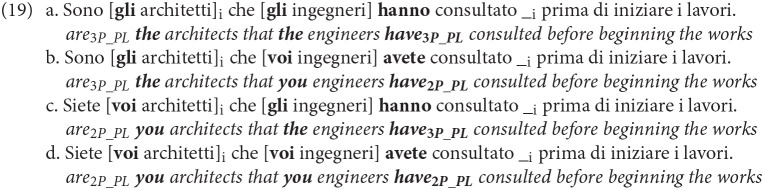


Hereafter, we will refer to (19).a as the *Art*_1_*-Art*_2_ matching condition, (19).b as *Art*_1_*-Pro*_2_ mismatch condition, (19).c as *Pro*_1_*-Art*_2_ mismatch condition and (19).d as *Pro*_1_*-Pro*_2_ matching condition. DP_1_ is the focalized object, DP_2_ the OC subject.

Overall, in off-line terms, similarity-based (section Similarity-Based Accounts), intervention-based (section Intervention-Based Accounts) and *top-down Minimalist* (based on FRC, section *top-down* (Left-Right) Minimalist Derivations) models would all predict that matching conditions (*Art*_1_*-Art*_2_ and *Pro*_1_*-Pro*_2_) should be more difficult than the mismatching conditions (*Pro*_1_*-Art*_2_ and *Art*_1_*-Pro*_2_). The *top-down* model, in particular, would predict a facilitation under the mismatch condition due to the distinct cued features at the verbal predicate [higher *dF* coefficient in the FRC, as expressed in (13)]. By default (hypothesis H1 in Table 2), as discussed in section Pronouns as Determiners and Agreement, any feature mismatch could help, hence the facilitation should be similar for both *Pro*_1_*-Art*_2_ and *Art*_1_*-Pro*_2_ conditions. However, according to the 1st and 2nd person anchoring hypothesis (H2 in section Pronouns as Determiners and Agreement), a global facilitation for the *Art*_1_*-Pro*_2_ condition compared with the *Pro*_1_*-Art*_2_ is expected by the *top-down* model: retrieval of *Pro*_2_ due to the cued 2nd person should be favored (hypothesis H2 in Table 2). Similarly, a retrieval penalty due to 2nd person feature matching is expected under the *Pro*_1_*-Pro*_2_ condition. Notice that H2 does not change in any relevant sense the predictions of any other model except for the *top-down* one.

**Table 2:** Theory by theory overall (off-line) predictions on the paradigm (19).

**Condition**	**Art_**1**_-Art_**2**_**	**Pro_**1**_-Pro_**2**_**	**Art_**1**_-Pro_**2**_**	**Pro_**1**_-Art_**2**_**
Similarity-based prediction	Hard	Hard	Medium	Medium
Intervention-based prediction	Hard	Hard	Medium	Medium
Top-down prediction (FRC)—H1	Hard	Hard	Medium	Medium
Top-down prediction (FRC)—H2	Hard	Hardest	Medium	Hard
Memory-load prediction—A1	Hard	Hard	Hard	Hard
Memory-load prediction—A2	Harder	Hard	Hard	Harder
Memory-load prediction—A3	Hard	Harder	Harder	Hard
ACT-R-based prediction	Hard	Hard	Hard	Hard

As for *memory-load* hypotheses (section *memory-load* Accounts), *Pro* condition has never been discussed previously, so the model here needs further assumptions: on the one hand, we might expect *Pro* and *Art* conditions to be referentially similar; under this assumption (A1 in Table 2) no difference would be predicted whatsoever in the paradigm. If *Pro* and *Art* are assumed to be referentially different, either *Art* turns out to be less accessible than *Pro* (A2 assumption in Table 2), then we should expect an extra encoding cost for *Art* and a related complexity signature for retrieving the focalized object in the *Art*_2_ condition, or *Pro* is less accessible than *Art* (A3 assumption in Table 2), then a greater effort should be paid in the *Pro*_2_ condition.

From a different perspective, ACT-R based approaches (section ACT-R-Based Predictions) would predict no difference across conditions since the distance between the focalized DP_2_ and the OC predicate is always the same and no cue can help in retrieving/reactivating the focalized object.

In the end the retrieval/intervention cost predictions for all the models can be summarized in the table below [consider “hard” to be the baseline, based on the evidence discussed in section Processing Object Clefts (OCs)]:

In terms of on-line predictions, costs at the VERB regions should be proportional to the predictions expressed in Table 2. Moreover, *memory-load* and *top-down Minimalist* models also predict specific encoding costs that should have an effect in terms of on-line measures at the related DP_1_, DP_2_, predicate and PP final regions: according to Gibson (2000), the verb introduces an event referent (+1), while the integration cost must be calculated as crossing the verbal event (+1) and the cost of the intervening nominal referents (+1 in Gibson, 2000). According to the *top-down* approach, the predicate introduces two relevant features, a temporal index T and V predicate, in conformity with the DP encoding hypothesis: *Art* should have an encoding cost of 2, an index *D* and a *N* predicate (section Pronouns as Determiners and Agreement). We expect *Pro* to pay an extra encoding cost due to the out of the blue inclusion of a speech act participant into the N predicate (+1) as suggested in section Pronouns as Determiners and Agreement; as result, *Pro* condition would cost globally 3 units. Taking *Art* = 2 and *Pro* = 3 as encoding cost baseline, both for *memory-load* and *top-down* models, only the *top-down* model predicts an extra cost for any duplicated category (+1 for *N* in DP_2_) and for a referential mismatch (+1 for II person matching at DP_2_ in *Pro*_1_*-Pro*_2_ condition).

Under these assumptions, we can compare on-line predictions at the relevant segments^16^ (Table 3).

**Table 3:** On-line predictions on the paradigm (19); at the verb segment (encoding+retrieval).

	**Condition**	**DP_**1**_**	**DP_**2**_**	**Verb**	**PP**
Art_1_-Art_2_	Memory-load prediction	2	2	4 (1+3)	4
	Top-down prediction	2	3	3.43 [2+log(27)]	5
Art_1_-Pro_2_	Memory-load prediction	2	3	5 (1+4)	4
	Top-down prediction	2	4	3.38 [2+log(24)]	5
Pro_1_-Art_2_	Memory-load prediction	3	2	4 (1+3)	4
	Top-down prediction	3	3	3.43 [2+log(27)]	5
Pro_1_-Pro_2_	Memory-load prediction	3	3	5 (1+4)	4
	Top-down prediction	3	5	3.68 [2+log(48)]	5

Crucially, the predicted cost at DP_2_ is different since the *top-down* model, and not the *memory-load* one, predicts an extra encoding cost which is proportional to the number of matching features (and consequently to the necessity of updating an expectation, also in terms of speech act participants, otherwise salient, hence “free”). Moreover, at the verb segment, the predictions of the two models differ: the *memory-load* model predicts major efforts in the *Pro*_2_condition, while the *top-down* model expects milder differences and essentially a penalty to be paid for the *Pro*_1_*-Pro*_2_ condition. Under H2 (i.e., only 1st/2nd person features are cued by the verb), also a mild facilitation for the *Art*_1_*-Pro*_2_ would be expected by the *top-down* model as compared to the *Art*_1_*-Art*_2_ and *Pro*_1_*-Art*_2_ conditions. Under H1 (i.e., all person features count as distinct cues), also *Pro*_1_*-Art*_2_ would benefit by the cued feature mismatch.

## Materials and Methods

### Ethics Statement

The experiment was approved by the Ethics Committee of the “Dipartimento di Scienze del Sistema Nervoso e del Comportamento” of the University of Pavia. A written informed consent was obtained from the participants of this study.

### Participants

Fifty-three participants (age range 20–52, *M* = 34.57, SD = 8.09, 28 female; all speakers of center-north Italian variety) voluntarily signed up for the acceptability study (experiment 1).

A different sample of 33 Italian native speakers of the same Italian variety (age range = 19–35; 18 female) took part in the eye-tracking study (experiment 2). After the end of each experimental session, in experiment 2, we assessed the participants' Verbal Working Memory Capacity (WM) using a test (in Lewandowsky et al., 2010) that is a variant of the Sentence Span test originally designed by Daneman and Carpenter (1980). Participants were asked to carry out a dual task: they were presented with series of 3 to 8 statements, each followed by a consonant. Participants had 4 sec to judge whether the statement was True or False then they were asked to remember the series of consonants that was presented after each sentence. At the end of the series a question mark appeared, signaling participants to type in all the consonants presented. A score ranging from 0 to 1 is obtained, indicating the individuals verbal working memory capacity. The scoring procedure takes into account the length of the series and the accuracy to the Ture/False judgment (see Lewandowsky et al., 2010, for more details).

### Stimuli

We created 32 paradigms expressing the four possible conditions presented in (19), for a total of 128 items. DPs were introduced by articles and second person pronouns while keeping number (plural, in order to make all the oppositions sound) and gender (masculine) constant. The nouns within each sentence were balanced for (i) number of letters (DP_1_ = 8.86, SD = 1.46; DP_2_ = 8.96, SD = 1.95; *t* < 1), (ii) logarithmic frequency (based on Repubblica corpus, Baroni et al., 2004) of nominal items (DP_1_ = 7,66, SD = 1.68; DP_2_ = 7.58, SD = 1.95; *t* < 1), and (iii) concreteness and imageability (all were concrete nouns referring to professions). The plural masculine article in Italian is sensitive to the beginning of the following noun: when a vowel is present “gli” is used (“gli architetti,” the architects vs. “i giorni,” the days). We used “gli” to maximize the length of the determiner and match the length of the pronoun (“voi,” you) whenever possible, but in most cases (22/32), due to semantic congruity and to keep DP frequency comparable within sentences we used “i” determiner for nouns beginning with a consonant. Experimental lists included 32 critical items (eight per condition) and 112 fillers. As in previous experiments, we did not include any relevant context. Fillers included 64 declarative sentences, half of them perfectly grammatical and the other half presenting a subject-verb number agreement error; other 48 fillers were questions with various degrees of acceptability, ranging from perfect grammatical *wh*- long distance question (“what do you think that John saw?”) to violation of locality (“what do you wonder who see?”). Both experiments used these materials.

A Latin square design was used to counterbalance conditions across experimental lists, in a way that in each experiment participants were exposed to one only version of each paradigm, and each item within paradigms was presented to an almost equal number of subjects across lists.

### Procedure

For experiment 1 (acceptability judgment), a web-based questionnaire was created using Osucre open source software (Van Acker, 2007). The experimental lists were presented one at a time on a single line and participants were asked to judge each for acceptability on a 7-points Likert-scale.

For experiment 2 (eye-tracking), participants were individually tested in a dimly lit room. They sat in front of a 17 inches computer screen and kept their head on a chin rest so that the distance between the display and their eyes was 56 cm. They were instructed to read the sentences carefully, as they would have to answer one question following each sentence. Each trial (presented in pseudo-randomized order) consisted of a fixation cross appearing at the center of the screen for 1,500 ms, and was followed by the sentence, displayed on one single line. Participants could press the space bar to signal the reading completion, or the sentence display timed out after 20 sec. After the sentence display a second fixation cross appeared for 1,500 ms, just before the presentation of the question, that remained on screen until the Yes/No response. Half of the questions concerned the subject (e.g., “did the architects consult someone?”) the other half the object of the cleft (e.g., “did someone consult the engineers?”); half of the questions included a relevant PP (e.g., “did the architects consult someone after the meeting?”); 50% of the questions required a positive answer, 50% a negative one.

### Analysis

#### Data Acquisition and Pre-processing of Eye-Tracking Data

Eye-movements in experiment 2 were recorded with an Eyelink® 1000 system (SR-Research, Ottawa, CA), tracking the dominant eye and using the desktop mount configuration. Eye gaze was sampled at 1,000 Hz frequency. Consecutive fixations between 50 and 80 ms occurring at one character distance were grouped into one single fixation (1.35% of data). Fixations that were (a) shorter than 80 ms, (b) longer than 1,200 ms, (c) occurring within 20 ms from blink onset/offset, and (d) occurring outside sentence boundaries were excluded from the analyses (overall rejection rate 3.16%). Four participant were excluded on the basis of their performance on the comprehension questions (<60%), leaving 29 participants in the analyzed dataset.

Four canonical reading time measures (Rayner, 1998) were computed. *First Fixation* (FF) and *Gaze Duration* (GD) were defined as the time (ms) spent on each region when participants entered it (from the left side) for the first time: FF was the duration of the very first fixation only, whereas GD was defined as the time spent from the first time entering the region to the first time leaving it, to the right or to the left. Words in the sentences could be fixated after a regression (i.e., entering the region from the right) and the time spent in a region entering it from the right was defined as *Second Pass* (SP) reading time. Total duration Time (TT) was the total time spent on a given region.

We also analyzed regression data: a regression event occurred when participants looked back in the sentence. For each regression event, we determined the region from where the eye left (*R-from*), and the region where the gaze landed (*R-in*).

Sentences consisted of six different regions: BE, DP_1_, C, DP_2_, VERB, SPILLOVER. Regression analyses were carried out for each region, when the number of observations was sufficient for carrying out statistics. In particular, only Total Duration and Second Pass could be computed for BE and C regions.

#### Statistics

Results were analyzed using linear mixed-effects regression models (Baayen et al., 2008), using lme4 package (version 1.1.21) in R environment (R Core Team, 2018). Mixed models are widely used in eye-movements research (e.g., Staub, 2010; Kuperman and Van Dyke, 2011) as they conveniently handle imbalanced designs and missing values, typically occurring with eye-tracking data.

Linear mixed models were used for acceptability judgments of Experiment 1 and Reading Times in Experiment 2. The dependent variables for the linear mixed models were the single trials acceptability scores (ranging from 1 to 7) for Experiment 1, and the log transformed Reading Times for Experiment 2. Log transformation was needed because the distribution of times and the models' residuals were far from the normal distribution. For the analysis of regressions in Experiment 2 the dependent variable was binary (0,1) depending on whether a regression event was or was not recorded, and data were analyzed by fitting generalized mixed models using the logit response function (e.g., Jaeger, 2008)—as it was done for the Accuracy data on the comprehension question of Experiment 2.

In Experiment 1 we used the “maximal” random structure allowing for by-subject and by-item intercept adjustments and by-subject slopes adjustments for DP_1_ by DP_2_ interaction. However, the maximal random structure of the models for Experiment 2 (where the number of participants was lower) often caused convergence issues. We therefore adopted a random structure chosen on grounds of feasibility (Matuschek et al., 2017). For reading measures (FF, GD, TT, and SP) the random structure was allowed by-subject and by-item intercept adjustments, and by-subject slope adjustments of DP_1_ and DP_2_. For regression measures the random structure was initially “minimal,” but, when possible, the model used to report the final estimates and the contrasts between conditions had the same structure used for the reading measures.

To evaluate the presence of significant main effects and interactions we used likelihood ratio tests (LRT) comparing the fit of (nested) models of increasing complexity (e.g., [factor A + B] compared to [factor A + B + AxB]). Tables in the Results section describe the outcome of the LRTs, reporting the value of chi square and the level of significance. In the analysis of Experiment 2 the models were further specified: the Null model consisted of the effect of Trial Order alone, and Trial Order was kept in all subsequent models. The contribution to the model fit was assessed for the factors DP_1_ (Art, Pro), DP_2_ (Art, Pro), and WM (continuous) and their interactions. To make the main effects interpretable as in standard ANOVAs we adopted contrast coding for categorical factors, while continuous predictors were z-centered around the mean (e.g., Levy et al., 2013). The effects of the factors of interest and their interactions are further described in the text and in the figures providing the size of the effect in the response measure (i.e., back transforming log(ms), in ms, and log odds into probability) using functions in the emmeans package (Lenth et al., 2019).

## Results

### Off-Line Results (Acceptability Judgment and Accuracy in Comprehension Questions)

**Figure 1:**
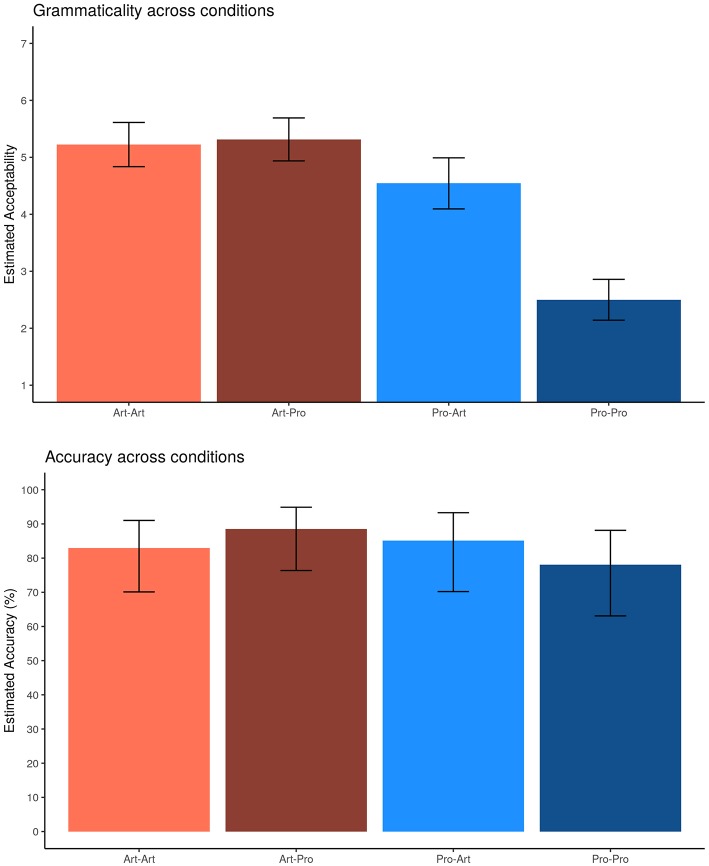
Offline results: estimated acceptability (7-point Likert scale) judgments (experiment 1) and accuracy (%) in answering comprehension questions (experiment 2). Error bars represent 95% confidence intervals around the models' estimates.

We compare here (Figure 1) the results of the offline data gathered from both experiments: the acceptability rate of experiment 1 and the accuracy in answering comprehension questions after eyetracking (experiment 2).

Summarizing the acceptability data collected in Experiment 1, we obtained the following pattern^17^:

*Art*_1_*-Pro*_2_ (*M* = 5.31, SE = 0.19) ≥ *Art*_1_*-Art*_2_ (*M* = 5.22, SE = 0.19) > *Pro*_1_*-Art*_2_ (*M* = 4.54, SE = 0.22) > *Pro*_1_*-Pro*_2_ (*M* = 2.50, SE = 0.18).

The analysis of Experiment 1 LRTs showed that judgments were influenced by the interaction between DP_1_ and DP_2_ (Table 4) showing that when DP_1_ was *Art* the subject type had no effect on acceptability (*Art*_1_*-Art*_2_ = 5.22; *Art*_1_*-Pro*_2_ = 5.31; *t* = −1.04), whereas when DP_1_ was introduced by a pronoun, the effect of subject type was sensible being the matching condition much less acceptable (*Pro*_1_*-Art*_2_ = 4.54; *Pro*_1_*-Pro*_2_ = 2.50; *t* = −9.28, *p* < 0.001).

**Table 4:** Acceptability and Accuracy effects depending on DP_1_-DP_2_ types.

	**χ^2^**	***p***
**Acceptability**		
DP_1_ type	**22.09**	**<0.001**
+ DP_2_ type	**5.49**	**<0.01**
+ DP_1_:DP_2_	**51.85**	**<0.001**
**Accuracy**		
DP_1_ type	0.69	
+ DP_2_ type	0.00	
+ DP_1_:DP_2_	3.76	<0.1

*Bold values are the significant ones*.

A similar numerical pattern indicating that *Art*_1_*-Pro*_2_ is better than *Pro*_1_*-Pro*_2_ is revealed in comprehension (while *Pro*_1_*-Art*_2_ ≥ *Art*_1_*-Art*_2_ is non-significant):

*Art*_1_*-Pro*_2_ (*M* = 88.6%) ≥ *Pro*_1_*-Art*_2_ (*M* = 85.1%) ≥ *Art*_1_*-Art*_2_ (83%) ≥ *Pro*_1_*-Pro*_2_ (78.1%).

The marginally significant interaction pointed to the effect of DP_1_ when DP_2_ is *Pro*, which describes the marginally significant difference between *Art*_1_*-Pro*_2_ and *Pro*_1_*-Pro*_2_ (+10.5%, *z* = 1.70, *p* < 0.1).

### On-Line Results (Eye Tracking Data, Experiment 2)

#### Reading Times

##### First fixation (FF)

**Figure 2:**
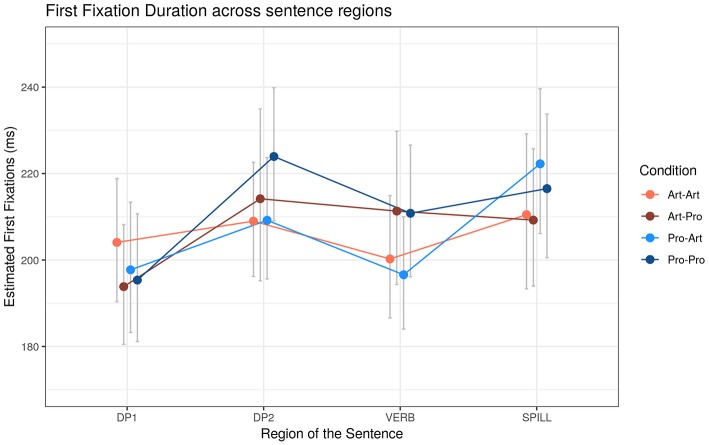
Estimated First Fixation (FF) reading times (ms) across sentence regions. Error bars represent 95% confidence intervals around the models' estimates.

Due to the small number of observations for FFs in BE (*n* = 115) and in C (*n* = 307), models did not converge and the results from these regions were omitted (Figure 2). Analyses (Table 5) revealed a main effect of DP_2_ type, indicating longer FFs for *Pro*_2_, while reading DP_2_ (+10 ms, *t* = 2.06, *p* < 0.05) and VERB (+10 ms, *t* = 2.39, *p* < 0.05) regions. Upon reading SPILL region, FFs were slightly longer with *Pro*_1_ (+9 ms, *t* = 1.98, *p* < 0.1).

**Table 5:** First Fixation (FF) effects depending on the DP_1_-DP_2_ types and Working Memory (WM).

	**Regions**											
**First fixation times**	**BE**		**DP_1_**		**C**		**DP_2_**		**Verb**		**Spill**	
	**χ^2^**	***p***	**χ^2^**	***p***	**χ^2^**	***p***	**χ^2^**	***p***	**χ^2^**	***p***	**χ^2^**	***p***
**Null (trial order)**												
+ DP_1_ type							1.21				**3.92**	**<0.05**
+ DP_2_ type			2.14				**4.98**	**<0.05**	**6.55**	**<0.05**		
+ DP_1_:DP_2_												
+ WM			1.29				**4.37**	**<0.05**	**8.58**	**<0.01**	**6.41**	**<0.05**
+ DP_1_:WM												
+ DP_2_:WM									2.08			
+ DP_1_:DP_2_:WM									1.32			

*χ^2^ <1 are omitted*.

*Bold values are the significant ones*.

Considering the effect of WM, it was significant across the last three regions: FFs were generally shorter for Higher WM participants (β in DP_2_: −0.038, *t* = −1.95, *p* < 0.1; β in VERB: −0.070, *t* = −3.44, *p* < 0.01; β in SPILL: −0.056, *t* = −2.51, *p* < 0.05).

##### Gaze duration (GD)

**Figure 3:**
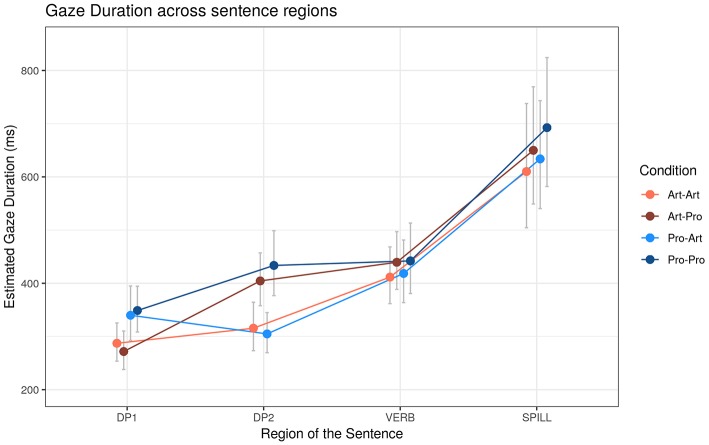
Estimated Gaze Duration (GD) reading times (ms) across sentence regions. Error bars represent 95% confidence intervals around the models' estimates.

Due to the small number of observations for GD in BE and in C, models did not converge and the results from these regions were omitted (Figure 3). In the analysis of GD (Table 6), a main effect of DP_1_ type in DP_1_, and a main effect of DP_2_ type in DP_2_, are significant. In DP_1_ region, *Pro*_1_ triggered longer GD compared to *Art*_1_ [+64 ms, *t* = 6.20, *p* < 0.001]. In DP_2_ region longer GD occurred with DP_2_ [DP_2_ region: +108 ms, *t* = 7.28, *p* < 0.001]. A marginal indication of a significant main effect of DP_2_ emerged also in the VERB region, showing a slow down to *Pro*_2_ [+26 ms, *t* = 1.86, *p* < 0.1].

**Table 6:** Gaze Duration (GD) effects depending on DP_1_-DP_2_ type and Working Memory (WM).

	**Regions**											
**Gaze duration times**	**BE**		**DP_1_**		**C**		**DP_2_**		**Verb**		**Spill**	
	**χ^2^**	***p***	**χ^2^**	***p***	**χ^2^**	***p***	**χ^2^**	***p***	**χ^2^**	***p***	**χ^2^**	***p***
**Null (trial order)**												
+ DP_1_ type			**30.1**	**<0.001**							1.6	
+ DP_2_ type							**32.09**	**<0.001**	3.42	<0.1	2.93	<0.1
+ DP_1_:DP_2_			1.11				2.42					
+ WM			**11.80**	**<0.001**			**8.12**	**<0.01**	17.1	**<0.001**	12.59	**<0.001**
+ DP_1_:WM												
+ DP_2_:WM							1.93		1.01			
+ DP_1_:DP_2_:WM							1.13					

*Bold values are the significant ones*.

The effect of WM, indicating shorter GD for participants with higher WM, was robust across regions: in DP_1_ (−0.115, *t* = −3.45, *p* < 0.001), DP_2_ (β = −0.097, *t* = −2.77, *p* < 0.01), VERB (β = −0.179, *t* = −4.37, *p* < 0.001), and SPILL (β = −0.181, *t* = −3.66, *p* < 0.001).

##### Total time duration (TT)

**Figure 4:**
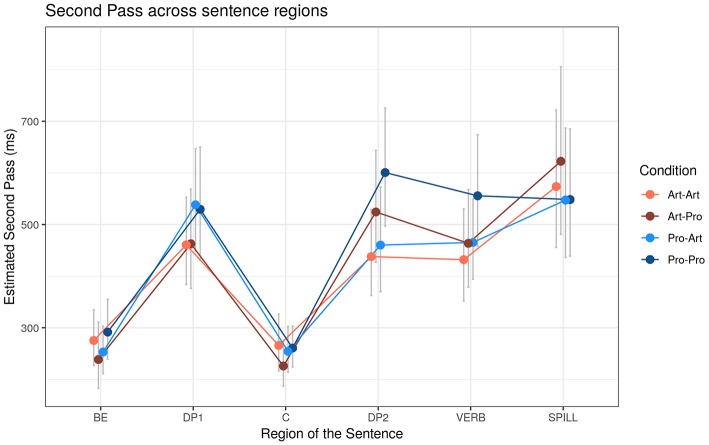
Estimated Total reading Times (TT) (ms) across sentence regions. Error bars represent 95% confidence intervals around the models' estimates.

As for TT (Figure 4), effects of the experimental factors were found on all Regions except for SPILL (Table 7).

**Table 7:** Total Time (TT) effects depending on the DP_1_-DP_2_ type and Working Memory (WM).

	**Regions**											
**Total reading times**	**BE**		**DP_1_**		**C**		**DP_2_**		**Verb**		**Spill**	
	**χ^2^**	***p***	**χ^2^**	***p***	**χ^2^**	***p***	**χ^2^**	***p***	**χ^2^**	***p***	**χ^2^**	***p***
**Null (trial order)**												
+ DP_1_ type	1.52		**28.22**	**<0.001**	2.84	<0.1	1.04		2.34			
+ DP_2_ type							**19.39**	**<0.001**	2.77	<0.1		
+ DP_1_:DP_2_	**4.37**	**<0.05**			1.58		**8.95**	**<0.01**	**6.33**	**<0.05**	1.32	
+ WM	3.53	<0.1			**3.86**	**<0.05**			1.21			
+ DP_1_:WM												
+ DP_2_:WM			3.60	<0.1			1.90					
+ DP_1_:DP_2_:WM												

*Bold values are the significant ones*.

In BE the significant interaction between DP types was due to the slow down (+43 ms, *t* = 1.84, *p* < 0.1) to *Pro*_1_ compared to *Art*_1_ that resulted marginally significant on the pairwise contrasts (*t* = 1.84, *p* < 0.1) in the *Pro*_2_ condition and not in *Art*_2_ (+2 ms, *t* < 1). In DP_1_, reading *Pro*_1_ took longer than *Art*_1_ (+168 ms, *t* = +6.71, *p* < 0.001), and TT in this region is further modulated by the interaction between DP_2_ type and WM: the facilitatory effect of WM was stronger (Δβ = −0.085, *t* = −1.90, *p* < 0.1) when DP_2_ was *Art* (β = −0.07) rather than *Pro* (β = 0.01). In DP_2_ region, the effect of DP_2_ (longer *Pro*_2_ compared to Art_2_: +183 ms, *t* = +5.41, *p* < 0.001) was further modulated by DP_1_ type: when DP_2_ was *Art*, no effect of DP_1_ emerged (−20 ms, *t* < 1), while when DP_2_ was *Pro*, longer total times were observed for *Pro*_1_–*Pro*_2_ compared to *Art*_1_–*Pro*_2_ (+137 ms, *t* = +3.31, *p* < 0.01). In the VERB region, as in the previous region, the interaction between DP types revealed that the effect of DP_1_ was present in the *Pro*_2_ condition–longer TT for *Pro*_1_–*Pro*_2_ compared to *Art*_1_–*Pro*_2_ (+83 ms, *t* = +2.35, *p* < 0.05)–and not in the *Art*_2_ condition (−17 ms, *t* < 1).

Total times spent in BE and C were globally influenced by WM [in BE: β = −0.095, *t* = −1.99, *p* < 0.1; in C: β = −0.064, *t* = −1.73, *p* < 0.1].

##### Second pass duration (SP)

**Figure 5:**
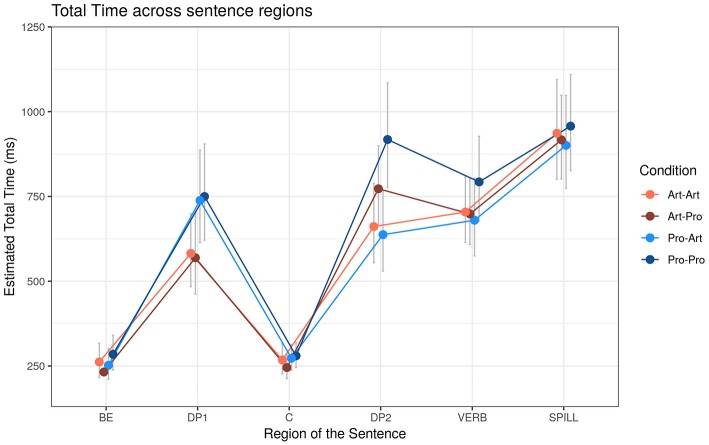
Estimated Second Pass (SP) reading times (ms) across sentence regions. Error bars represent 95% confidence intervals around the models' estimates.

The presence of a pronominal restriction in DP_1_ and DP_2_ was sufficient to cause longer SP reading times in both DP_1_ and DP_2_ regions (Figure 5, Table 8): SP in DP_1_ were longer for *Pro*_1_ (+73 ms, *t* = +2.89, *p* < 0.01), and, similarly, in DP_2_ region SP were longer for *Pro*_2_ (+111 ms, *t* = +3.21, *p* < 0.01). In DP_2_, however, participants show longer SP in the *Pro*_1_ condition compared to *Art*_1_ condition (+45 ms, *t* = +1.75, *p* < 0.1).

**Table 8:** Second Pass (SP) reading times effects depending on the DP_1_-DP_2_ type and Working Memory (WM).

	**Regions**											
**Second pass reading times**	**BE**		**DP_1_**		**C**		**DP_2_**		**Verb**		**Spill**	
	**χ^2^**	***p***	**χ^2^**	***p***	**χ^2^**	***p***	**χ^2^**	***p***	**χ^2^**	***p***	**χ^2^**	***p***
**Null (trial order)**												
+ DP_1_ type			**6.68**	**<0.01**			**3.88**	**<0.05**	**6.33**	**<0.05**	1.64	
+ DP_2_ type							**9.70**	**<0.01**	**3.89**	**<0.05**		
+ DP_1_:DP_2_	**5.06**	**<0.05**			2.73	<0.1						
+ WM	1.89				2.21				2.66			
+ DP_1_:WM			1.51				2.13		2.37			
+ DP_2_:WM			2.08				1.27		2.47			
+ DP_1_:DP_2_:WM							2.13		**3.82**	**=0.05**		

*χ^2^ <1 are omitted*.

*Bold values are the significant ones*.

In the VERB region, a three-ways interaction emerged, showing that *Pro* generally causes a slowdown in SP (*Pro*_2_ condition: +52 ms, *t* = 1.78, *p* < 0.1; *Pro*_1_condition: +57 ms, *t* = 2.01, *p* < 0.05), and that the effect of WM (faster reading times for higher WM) was much stronger in *Pro*_1_*-Art*_2_ (β = −0.214), compared to the other three conditions [*Pro*_1_*-Pro*_2_ β = 0.025, Δβ = −0.239, *t* = −2.75, *p* < 0.05; *Art*_1_*-Art*_2_ β = 0.036, Δβ = −0.250, *t* = −3.04, *p* < 0.05; *Art*_1_*-Pro*_2_ β = 0.018, Δβ = −0.232, *t* = −2.45, *p* < 0.1].

In the end, SP in BE showed a significant DP_1_ by DP_2_ interaction. The time spent re-reading BE was not affected by DP_1_ type when DP_2_ was *Art* (18 ms, *t* < 1), while for *Pro*_2_ the difference between *Pro*_1_ and *Art*_1_ was consistent (+49 ms, *t* = 1.99, *p* = 0.05).

#### Regressions

We first assessed the likelihood of performing a regression From each region, independently of the experimental factors, but distinguishing between regressions in first pass and overall regressions. The probability of performing a regression was largest from the rightmost region SPILL (80%), followed by DP_2_ (46.8%, SPILL vs. DP_2_: *z* = +13.48^***^), VERB (29.9%, DP_2_ vs. VERB: *z* = +7.00^***^), DP_1_ (23.3%, VERB vs. DP_1_: *z* = +3.15^*^), and C (19.8%, VERB vs. C: *z* = +4.26^***^).

Notably, the probability of performing a regression during first pass was considerably reduced for the VERB region (9.42%).

VERB first pass regressions were less likely than those occurring in DP_1_ (13.75%, *z* = −2.79^*^) or DP_2_ (19.30%, *z* = −6.02^***^), suggesting that processing difficulties at the VERB may be different from those at DP regions. In particular, the integration efforts at DP_1_ or DP_2_ trigger immediate regressions, while encoding/retrieval costs do not immediately lead to a regressive saccade, but rather require more reading time.

All in all, the overall probability in regressions pattern suggests that the DP_2_ region is particularly hard to process: upon reading this region, being it the first time or not, participants need to go back in the sentence to retrieve additional information.

##### Regressions from regions (R-from)

**Figure 6:**
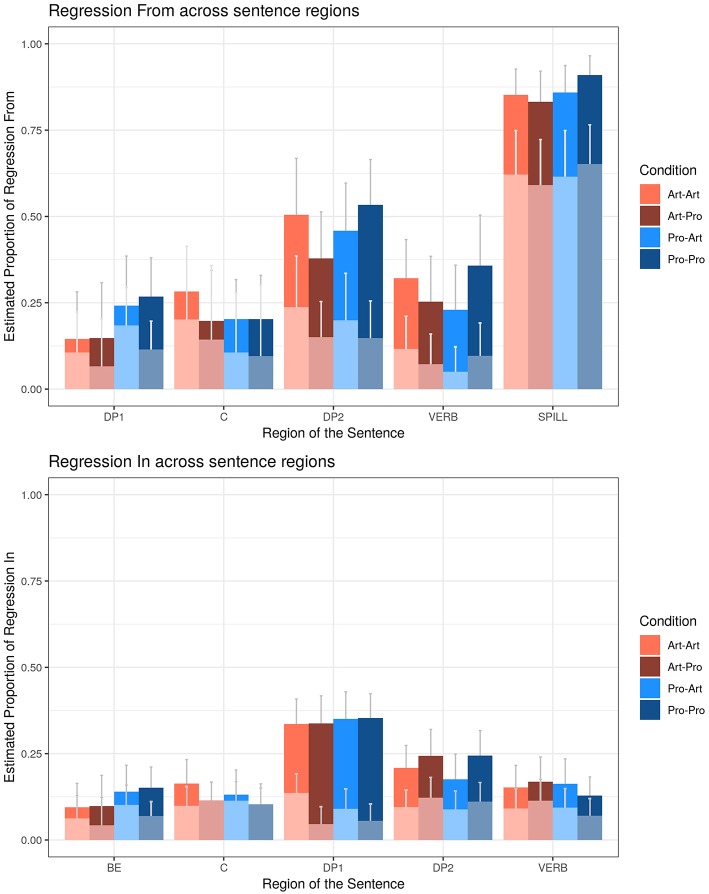
Estimated first (light shades) and total (full colors) regression probabilities (%) In and From the regions of interest. Error bars represent 95% confidence intervals around the models' estimates.

We first evaluated the probability of making a R-from each ROI on the total number of fixations on each region (Figure 6, Table 9). A main effect of DP_1_ type in DP_1_ region, showed that regressions from DP_1_ were more likely for DP_1_ *Pro* (*Pro*_1_ vs. *Art*_1_: +0.11, *z* = 2.84, *p* < 0.01). In the pattern of Regressions from VERB and DP_2_ regions significant interactions between DP_1_ and DP_2_ emerged. When DP_1_ was *Pro*, more regressions out of VERB were associated to *Pro*_2_ (*Pro*_2_ vs. *Art*_2_: +0.11, *z* = 2.06, *p* < 0.05), while when DP_1_ was *Art* the pattern was numerically opposite (*Pro*_2_vs. *Art*_2_: −0.06, *z* = −1.30, ns). Considering a *post hoc* mismatching vs. matching pairwise comparison, in addition to DP type comparison, we observe that mismatching conditions are associated to a much smaller regression probability from this region (*mismatching* vs. *matching*: −0.10, *z* = −2.754, *p* = 0.005). As for Regressions from DP_2_ the interaction was explained by a similar pattern: here, though, when DP_1_ was *Pro*, the higher proportion of regressions out for *Pro*_2_was not significant (*Pro*_2_vs. *Art*_2_: +0.07, *z* = 1.45, ns), while when DP_1_ was *Art* a higher likelihood of making a regression was observed for DP_2_ *Art* (*Art*_2_ vs. *Pro*_2_: +0.13, *z* = 2.25, *p* < 0.05).

**Table 9:** Regression from (R-from) depending on the DP_1_-DP_2_ types and Working Memory (WM).

**Regression from**	**DP1 (*****N*** **=** **198)**		**DP2 (*****N*** **=** **396)**		**Verb (*****N*** **=** **300)**		**Spill (*****N*** **=** **708)**	
	**χ^2^**	***p***	**χ^2^**	***p***	**χ^2^**	***p***	**χ^2^**	***p***
Null (trial order)								
+ DP_1_ type *_*fR*−*from*_*	**6.16**	**<0.05**	2.19		<1		1.64	
+ DP_2_ type *_*fR*−*from*_*	_*2.63*_		_*8.67*_	_<*0.01*_	1.29			
+ DP_1_:DP_2_ *_*fR*−*from*_*	_*1.93*_		**7.68**	**<0.05**	**7.80**_*4.72*_	**<0.01**_<*0.05*_		
+ WM *_*fR*−*from*_*	3.31 _*6.72*_	<0.1 _<*0.01*_			**4.87**	**<** **0.05**	_*1.31*_	
+ DP_1_:WM *_*fR*−*from*_*								
+ DP_2_:WM *_*fR*−*from*_*	3.48	<0.1	**4.10**	**<0.05**				
+ DP_1_:DP_2_:WM *_*fR*−*from*_*	1.81		2.38				_*2.89*_	_<*0.1*_

*First Regression from are indicated (_fR−from_) under the total R-From estimate. χ^2^ <1 are omitted*.

*Bold values are the significant ones*.

*Italics indicate the fR–from measures*.

The effect of WM, occurring in VERB (β = +0.403, *t* = 2.32, *p* < 0.05) and SPILL (β = +0.723, *t* = 2.58, *p* < 0.01) shows that participants with higher WM were more likely to make a regression out of these regions, whereas the marginally significant effect of WM in DP_1_ had the opposite direction (β = −0.375, −1.82, *p* < 0.1), suggesting that low WM participants performed a regression much earlier on. In DP_2_, the interaction between DP_2_ type and WM was due to the more positive (Δβ = +0.31, *z* = +1.97, *p* < 0.05) slope of WM for *Pro*_2_ (β = +.35) compared to *Art*_2_ (β = +0.04).

Then we evaluated Regressions in first pass, considering the proportion of regressions from each region on the number of fixations made in each region during first pass only. Regressions out of C were more likely for *Art*_1_ (+0.08, *z* = 1.93, *p* = 0.05). First pass Regressions from DP_2_ were more likely for *Art*_2_ (+0.12, *z* = 3.04, *p* < 0.05). At the VERB, the DP_1_ by DP_2_ interaction was due to a larger number of regressions for *Art*_1_ compared to *Pro*_1_ (+0.07, *z* = 2.08, *p* < 0.05) when DP_2_ was *Art*, which was absent when DP_2_ was *Pro* (−0.02, *z* < 1, ns).

##### Regressions into regions (R-in)

We first evaluated the likelihood of making a R-in for each ROI on the total number of Regressions events (Figure 6, Table 10). Regressions in C and DP_2_ were affected by the main effect of DP_2_ type: in C regressions were more likely for *Art*_2_ (+0.05, *z* = 2.84, *p* < 0.01), while in *DP*_2_ regressions were more likely for *Pro*_2_ (+0.06, *z* = 3.00, *p* < 0.01). The effect of WM had a negative slope in BE (β = −0.444, *z* = −4.01, *p* < 0.001) and in C (β = −0.257, *z* = −2.32, *p* < 0.05), suggesting that regressions in these regions were more likely for low WM participants, while the effect of WM had more positive slopes in DP_1_ (β = 0.135, *z* = 2.09, *p* < 0.05), DP_2_ (β = 0.212, *z* = 2.55, *p* < 0.05) and VERB (β = +0.222, *z* = 2.46, *p* < 0.05), showing that higher WM participants directed their regressions toward these sentence regions.

**Table 10:** Regression In (R_in_) depending on the DP_1_-DP_2_ types and Working Memory (WM).

**Regression in**	**BE (*****N*** **=** **286)**		**DP1 (*****N*** **=** **534)**		**C (*****N*** **=** **243)**		**DP2 (*****N*** **=** **336)**		**C (*****N*** **=** **228)**	
	**χ^2^**	***p***	**χ^2^**	***p***	**χ^2^**	***p***	**χ^2^**	***p***	**χ^2^**	***p***
**Null (trial order)**										
+ DP_1_ type	3.00	<0.1								
+ DP_2_ type					**7.01**	**<0.01**	**8.35**	**<0.01**		
+ DP_1_:DP_2_					2.43				1.09	
+ WM	**13.42**	**<0.001**	**4.69**	**<0.05**	**5.90**	**<0.05**	**6.39**	**<0.05**	**5.60**	**<0.05**
+ DP_1_:WM			1.32		3.02	<0.1				
+ DP_2_:WM	1.90				1.20					
+ DP_1_:DP_2_:WM					2.02					

*χ^2^ <1 are omitted*.

*Bold values are the significant ones*.

#### Summary

Acceptability judgments showed that matching conditions are significantly different (*Art*_1_*-Art*_2_ better than *Pro*_1_*-Pro*_2_). *Art*_1_*-Art*_2_ matching condition results slightly less grammatical than *Art*_1_*-Pro*_2_ mismatching condition. The other *Pro*_1_*-Art*_2_ mismatch condition ranking below them and above *Pro*_1_*-Pro*_2_ matching condition, which is unquestionably considered rather ungrammatical.

Comprehension questions in the eye-tracking experiment, revealed only an interaction between DP types, showing a difference in accuracy between *Art*_1_*-Pro*_2_ and *Pro*_1_*-Pro*_2_, but overall, the experimental sample correctly (>85%) understood the sentences.

Concerning reading times, and looking at the global results we can summarize:

- *Art*_1_*-Art*_2_ matching condition constitute the processing baseline in all measures;
- *Art*_1_*-Pro*_2_ mismatch condition caused some slow-down, mainly at DP_2_, where *Pro* was present (GD, TT, and SP), and marginally at VERB (FF only);
- *Pro*_1_*-Art*_2_ mismatch condition caused some slow-down, but only on DP_1_ region (TT and SP);
- *Pro*_1_*-Pro*_2_ matching condition is numerically the most time consuming condition in all measures, and the numeric differences emerge as statistically consistent with the DP_1_ DP_2_ interaction in TT for DP_2_ and VERB regions.

About factors interaction:

- the interactions between DP_1_ and DP_2_ was found in DP_2_ and VERB regions for TT; in BE and C for SP; in the regressions from DP_2_ and VERB.
- the effect of DP_2_ type is overwhelming: *Pro*_2_ is problematic as revealed in FF, GD, TT, and SP both in DP_2_ and VERB regions;
- mismatching conditions (*Art*_1_*-Pro*_2_and *Pro*_1_*-Art*_2_), overall, are associated to a reduced probability to trigger regressions from DP2 and VERB regions.
- the effect of WM is very strong, and, interestingly, the slope of WM is not always negative (higher WM associated with faster reading or fewer regressions). It has a negative slope in reading times measures FF, GD, TT, and SP. However, it shows different effects on regression probability. Higher WM is associated to a larger proportion of regressions out of VERB and SPILL regions, but to fewer regressions out (during first pass) of earlier sentence regions like DP1. No major interaction between WM ^*^ DP_1_^*^ DP_2_ type is revealed.

In Table 11 a summary of the main results.

**Table 11:** Main results summarized.

	**Condition**	**Art_**1**_-Art_**2**_**	**Pro_**1**_-Pro_**2**_**	**Art_**1**_-Pro_**2**_**	**Pro_**1**_-Art_**2**_**
**Region**	**Measure**				
	Acceptability	Good	Bad	Good	Medium
	Comprehension	Good	Good	Good	Good
DP1 (focalized object)	First fixation	Baseline	Baseline	Baseline	Baseline
	Gaze	Baseline	Slower	Baseline	Slower
	Total	Baseline	Slower	Baseline	Slower
	Second pass	Baseline	Slower	Baseline	Slower
	Regressions from	Baseline	More	Baseline	More
	Regressions in	Baseline	Baseline	Baseline	Baseline
D2 (subject)	First fixation	Baseline	Slower	Slower	Baseline
	Gaze	Baseline	Slower	Slower	Baseline
	Total	Baseline	Slower	Mildly slower	Baseline
	Second pass	Baseline	Slower	Mildly slower	Baseline
	Regressions from	More	More	Baseline	Baseline
	Regressions in	Baseline	More	Baseline	Baseline
Verb	First fixation	Baseline	Slower	Slower	Baseline
	Gaze	Baseline	Slower	Baseline	Baseline
	Total	Baseline	Slower	Baseline	Baseline
	Second pass	Baseline	Slower	Baseline	Baseline
	Regressions from	Slightly more	More	Baseline	Baseline

## Discussion

Starting with off-line considerations, the results of both experiments consistently show that a lexically restricted second person pronoun (“you linguists”) is at least as hard as a restricted definite article (“the linguist”) across all conditions, hence any advantage of the bare pronominal DPs (“you”), revealed in previous experiments (Warren and Gibson, 2005, a.o.), is lost when a lexical restriction is present. This main result is consistent both with the intervention-based (Friedmann et al., 2009; Belletti and Rizzi, 2013) and with the similarity based prediction (Gordon et al., 2001). These models, however, fail to capture contrasts both in matching conditions (with *Art*_1_*-Art*_2_ condition “easier than” *Pro*_1_*-Pro*_2_ condition) and in mismatching ones (with *Pro*_1_*-Art*_2_ condition less acceptable than *Art*_1_*-Pro*_2_ condition). Both contrasts are predicted under the *memory-load* and *top-down* perspectives: *memory-load* approach (Gibson, 1998) can predict an extra cost in processing *Pro* conditions by relying on the absence of an appropriate context (assumption A3 in section Testing the Different Predictions), but it fails to predict the striking asymmetry found between *Art*_1_*-Pro*_2_ and *Pro*_1_*-Pro*_2_. This contrast is only correctly predicted by the *top-down* model: the acceptability pattern revealed a significant difference in these conditions (*Art*_1_*-Pro*_2_ better than both *Pro*_1_*-Art*_2_ and, even more robustly, *Pro*_1_*-Pro*_2_) is predicted by this model under the H2 hypothesis: 2nd person on the subject, in a mismatching condition, induces a facilitation better than 3rd person (section Testing the Different Predictions). Similarly, under the same hypothesis, only the *top-down* model predicts a major effort in processing the *Pro*_1_*-Pro*_2_ with respect to the *Art*_1_*-Art*_2_ condition due to 2nd pronominal matching feature. This model also predicts a milder advantage of the *Art*_1_*-Pro*_2_ condition with respect to the *Art*_1_*-Art*_2_ condition which is numerically present in accuracy but not significant. Generally the pattern across conditions is similar both in acceptability and in accuracy in comprehension questions after eye-tracking. However, the first pattern, but not the second (with the exception of *Pro*_1_*-Pro*_2_ vs. *Art*_1_*-Pro*_2_, again coherently with H2), results in statistically significant contrasts. Notice that the *Pro*_1_*-Pro*_2_ condition is considered nearly ungrammatical by the subjects. This clearly differentiate this condition from the others. Also in comprehension questions this condition leads to the worst performance, but such performance is still surprisingly high (78.1%). This indicates that the subjects correctly answer to questions posed on sentences that they consider unacceptable, suggesting a milder discriminative power of the accuracy measure with respect to acceptability judgments.

Due to a constant set of factors (i.e., distance between the focalized object and the predicate, same context and same cued-features for the focalized DP), ACT-R-based processing model (Lewis and Vasishth, 2005) flatly predicts no difference among any of the tested configurations suggesting that this model needs extra assumptions to account for the revealed asymmetries.

**Figure 7:**
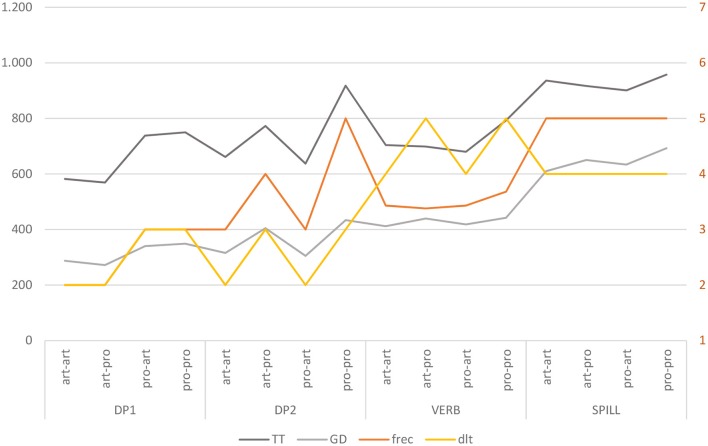
TT and GD estimates compared to FREC and DLT metrics.

Considering the online predictions (Figure 7), assuming an encoding cost penalty for the *Pro* conditions (hypothesis A3 in section Testing the Different Predictions), *memory-load* model becomes competitive in predicting GD reading times (*r* (14) = 0.65, *p* = 0.006) and (less robustly) and TT (*r* (14) = 0.53, *p* = 0.034).

Feature Retrieval and Encoding Cost (FREC) (FRC+FEC) however, with the very same assumptions on *Pro* encoding penalty and under the hypothesis that only 2nd person features on the subject are cued by the verb (H2 in section Testing the Different Predictions), correlates much more precisely both with GD (*r* (14) = 0.90, *p* < 0.001) and (even better) with TT (*r* (14) = 0.96, *p* < 0.001). Considering the nature of retrieval, the higher correlation with respect to the most comprehensive, latest, measure (i.e., TT) is expected.

As predicted under Bianchi (2003, 2006), Sigurdhsson (2004) and Elbourne (2005) analyses, the difficulty associated to the processing of the *Pro* condition is mostly due to the out of the blue presentation of the second person feature restricted by a *N* predicate. Therefore, the processing cost revealed at the DP regions where *Pro* occurred is likely due to encoding (need of postulating the salience of the relevant referents and update the common ground accordingly): this is revealed by longer reading times in late measures (GD, TT, and SP) comparable at both DPs regions under the *Pro* condition. In DP_2_ region, also an effect at the early FF measure was observed for *Pro*_1_*-Pro*_2_, suggesting an element of surprise as soon as the second restricted pronoun is encountered in the subject cleft position. A similar early effect, dependent on the presence of Pro_2_, is revealed also at the verb segment possibly indicating a “spillover effect” of the previous region processing or a retrieval difficulty. The spill-over interpretation only is supported by the fact that the DP_2_ effect disappears in later measures at the VERB segment (non-significant effect in GD and TT). On the other hand a retrieval problem in matching conditions is suggested by the first and total regression patterns leaving the VERB region: the significant *post hoc* pairwise comparison indicates less regressions from the verb in the mismatching conditions, possibly revealing a difficulty in retrieving the correct argument in the matching cases (especially with second person feature matching). Considering that the same effect is observed also at DP_2_, we should conclude that the retrieval interpretation is not the sole possible analysis.

This difficulty in the matching conditions is in line with the *top-down, similarity*-based and *intervention*-based predictions, but not with the *memory-load* ones.

Under the H2 hypothesis (2nd person feature on the subject cued by verb agreement morphology should facilitate the integration of the subject, while 3rd person default agreement should represent just a minor facilitation) the top-down model would predict an asymmetry also in the mismatching condition. However, no significant on-line evidence of a facilitation in retrieval is encountered for the *Art*_1_*-Pro*_2_ vs. *Pro*_1_*-Art*_2_ neither in terms of reading times at VERB, or regression probability. The on-line results then support hypothesis H1 (section Testing the Different Predictions), namely that both 2nd and 3rd feature mismatch on the VERB might facilitate retrieval (quicker FF), but *Pro*_2_ encoding penalty makes this effect hardly detectable. We agree with the reviewers suggesting that one way to tease apart the actual impact of the encoding penalty with respect to the retrieval effect would be to include in this experimental design the Subject Cleft condition where only encoding (and no retrieval penalty due to matching features to be re-merged) will be present. Another way to disentangle the two components would be to introduce a proper context, then removing the encoding penalty of *Pro* predicted under the assumption A3.

We can only suppose here that the encoding cost, responsible for the on-line slowdown revealed in the *Pro* conditions, could have been partially mitigated by the (mild) facilitation at retrieval in the mismatch *Art*_1_*-Pro*_2_ case, in the end producing an acceptability equivalence between the *Art*_1_*-Art*_2_ matching condition and the *Art*_1_*-Pro*_2_ mismatch condition. Everything being equal, removing the encoding cost, i.e., providing an appropriate context, we expect a difference *Art*_1_*-Art*_2_ vs. (worse than) *Art*_1_*-Pro*_2_ to appear, hence confirming the facilitation related to the usage of a deictic, mismatching, 2nd person feature in the intervening DP whose morphology is cued by the selecting verb (hence confirming the explanatory superiority of H2 over H1 also in on-line measures). Coherently with Staub (2010, p. 77-78), the complexity signatures at DP_2_ revealed (also) by a generally higher probability of making a regression from this region, especially in the matching conditions case (and especially in the *Pro*_1_*-Pro*_2_ case) could be interpreted as an indication of “something is wrong” (as in E–Z reader 10 model, Reichle et al., 2009) or, more precisely, an integration failure due to time out (c.f. Staub, 2010, p. 83) because of a context-update request.

To conclude, a final crucial intent of this study was to provide some new evidence for disentangling the (complex) relation between off-line and on-line performance measures. Given the off-line results gathered, first, we observed that acceptability judgments are more discriminative than accuracy in comprehension questions (though both generally correlates on the numerical patterns), second, FRC metrics, based on the *top-down* model, is the one making the closest predictions with respect to the pattern revealed across conditions. This suggests that the retrieval effort, at least in this context, is the best predictor of the overall acceptability and that, despite heavy encoding efforts (revealed by on-line measures), readers are fully rewarded by an adequate comprehension, revealed by accurate answers in all conditions.

As for the on-line data, again FRC+FEC (FREC) shows the best correlation with respect to the revealed “late” measures (GD and TT). Unexpected referents, introducing features that force a revision of the common ground assumptions are correctly predicted to affect performance by the FEC component at specific regions. These predictions crucially rely on a precise linguistic theory that takes into consideration the nature of the OC dependency and the relation between D, N types and person features. It is important to emphasize that no significant interaction between WM and our syntactic manipulation has been revealed: high WM participants simply show faster reading times and more regressive patterns compared to the low WM population across all conditions.

The actual usage of working memory during the processing of these specific constructions is still to be explored precisely. Nevertheless, we believe that the intuition that identical features that must be (re)merged within the active workspace are lighter to be processed than new ones or “similar” ones that must be kept distinct in memory (like an extra nominal restriction or an extra second person index) is worth further investigation: this idea is coherent with a “primed” active storage in which the “memory units” encoding a specific feature, being just activated would be more accessible than other units (on the line of ACT-R Lewis and Vasishth, 2005 intuition), while forcing a minimal diversification of a new pattern with respect to a pre-activated overlapping one has a considerably high cost.

## Ethics Statement

The experiment was approved by the Ethics Committee of the Dipartimento di Scienze del Sistema Nervoso e del Comportamento of the University of Pavia.

## Author Contributions

CC designed and directed the project, developed the theoretical top-down framework, the FR(E)C complexity measure, and performed the acceptability judgment experiment. PC performed the eye-tracking experiment and analyzed the results of both experiments. Both authors discussed the results and contributed to the final manuscript.

### Conflict of Interest Statement

The authors declare that the research was conducted in the absence of any commercial or financial relationships that could be construed as a potential conflict of interest.

## Footnotes

^1^In SR, the head is related to the subject position within the relative as in “the banker [that _ praised the barber ] …”.

^2^In this specific experiment, since “Ben”/“you” items are shorter than “the barber,” the discussed effects could be biased by the relative NP length. However, the same contrast is reported in other experiments [e.g., (Warren and Gibson, 2002), discussed later] where longer names are used, so we assume here that the relevant contrast is genuine.

^3^Other factors are assumed to induce a facilitation in processing this configuration: for instance animacy of the subject while the object is inanimate (Kidd et al., 2007), but see Belletti and Chesi (2014) for discussion.

^4^Belletti (2008) assumes that, in Subject Clefts, the Subject raises to CP to satisfy an EPP feature (hence the CP becomes a small clause in the sense of Stowell, 1983); this would create an intervention context in case the object or the indirect object would move across the subject position. As a consequence, only the subject can realize the new information focus, using the *vP* periphery of the matrix copula while the focalized (indirect) object moves to the CP peripheral FocP, where no EPP is present (exactly as a wh-item). In this position, it can only express contrastive/corrective (and not simply new information) focus.

^5^Thanks to Tessa Warren for sharing the original data. Differences in gray shades indicate statistically significant differences.

^6^Assuming an accessibility hierarchy (Ariel, 1990), the model postulates a discourse referent cost following this referentiality scale: (less accessible) definite description > proper names > referential pronoun (*he/she/him/her*) > deictic pronoun (*I/we* and *you*) (more accessible). The structural integration cost is proportional to the referent cost.

^7^The relevant opposition they propose is the one discussed in Munaro (1999):

(i) a. Con **che tosat** à-tu parlà? *with which boy did you speak?*
b. Avé-o parlà de **chi**?*have you spoken of whom?*
  Notice that the difference between the *wh*-items involved in this construction, *per se*, could be responsible for the different landing sites; the relevant comparison should involve a minimal pair of the kind “that” vs. “that boy” for which distinct positions are targeted according to the presence of the lexical restriction.

^8^Context-Free Grammar rules (Chomsky, 1965) and a Left Corner Parsing algorithm (Aho and Ullman, 1972) is assumed as well as various HPSG details (Pollard and Sag, 1994). Refer to Supplementary Materials section ACT-R-Based Model for technical details.

^9^We adapt here Stabler's (1996) Minimalist Grammar formalism: features associated to a lexical item are enclosed under squared brackets; categorial features are capital letters to the left of the lexical entry (e.g., “V” in [_V_ run …]), select features are ordered after a lexical entry and are prefixed by the equal sign (e.g., “=D” in [… run _= D_]).

^10^*S* indicates the subject (topic-related/EPP) functional position (see Bianchi and Chesi, 2014 for a discussion of the subject-related positions in this framework). Round brackets indicate optional features, i.e., *(S)* and *(Foc)* can be associated to *D* or not. The structure of the various DPs implements Longobardi's (1994) raising analysis. Retrieval from *M*(emory) will always preempts lexical insertion; *M* is a last-in-first-out memory. Angled brackets indicate unpronounced copies of an item. See the Supplementary Materials section Top-Down Minimalist model for technical details and the full derivation.

^11^The intent of this formula (compared to more complex and complete ones, Van Dyke, 2007; Chesi, 2017) is to highlight the factors that are necessary and sufficient to account for the minimal variations in the paradigm under discussion.

^12^Logarithmic function (i.e., log(FRC)) is provided for making this cost directly usable in on-line predictions at a comparable scale with respect to other metrics [e.g., (Gibson, 1998) or (Lewis and Vasishth, 2005)]. This is how the FRC is calculated, condition by condition (see Supplementary Materials section Top-Down Minimalist model for more details): D_1_-D_2_ = 9·3 = 27: 9 for retrieving D_2_, since *nF* = 2 (D and *N* count as one), *m* = 2 because two DPs are in memory at this time, and *dF* = 0 because no feature is cued by the verb distinguishing one DP from the other; 3 for retrieving D_1_, since *nF* = 2 (D and N are new), *m* = 1 and *dF* = 0; D_1_-N_2_ = 4·3 = 12: 4 for retrieving N_2_ (*nF* = 1, that is N, since D is contextually salient as we will see in section 0, *m* = 2, *dF* = 0), 2 for retrieving D_1_ (*nF* = 2, *m* = 1, *nF* = 0); D_1_-P_2_ = 2·3 = 6: 2 for retrieving P_2_ (*nF* = 1 even if deictic pronouns are contextually salient, the correct person must be retrieved, *m* = 2, *dF* = 1 since a distinct case on pronouns is cued by the verb), 3 for retrieving D_1_ (*nF* = 2, *m* = 1, *nF* = 0) N_1_-D_2_ = 9·2 = 18: 9 for retrieving D_2_ (*nF* = 2, since D and N are new, *m* = 2, *dF* = 0), 2 for retrieving N_1_ (*nF* = 1, *m* = 1, *nF* = 0); N_1_-N_2_ = 9·2 = 18: 9 for retrieving N_2_ (*nF* = 2 even though D should be contextually salient, being two proper names presents, the same D, i.e., a co-referential index, cannot be sufficient to distinguish them, then an extra cost must be paid here as in the D–D condition, *m* = 2, and *dF* = 0), 2 for retrieving N_1_ (*nF* = 1, just *N* is new since the determiner is now contextually salient and unique, *m* = 1 and *dF* = 0); N_1_-P_2_ = 2·2 = 4: 2 for retrieving P_2_ (*nF* = 1, *m* = 2, *dF* = 1); 2 for retrieving N_1_ (*nF* = 1, *m* = 1, *dF* = 0); P_1_-D_2_ = 9·2 = 18: 9 for retrieving D_2_ (*nF* = 2, *m* = 2, *dF* = 0); 2 for retrieving P_1_ (*nF* = 1, *m* = 1, *dF* = 0); P_1_-N_2_ = 4·2 = 8: 4 for retrieving N_2_ (*nF* = 1, *m* = 2, *dF* = 1); 2 for retrieving P_1_ (*nF* = 1, *m* = 1, *dF* = 0); P_1_-P_2_ = 2·2 = 4: 2 for retrieving P_2_ (*nF* = 1, *m* = 2, *dF* = 1); 2 for retrieving P_1_ (*nF* = 1, *m* = 1, *dF* = 0);

^13^Intervention-based approach would predict a specific facilitation for the 1st/2nd vs. 3rd person mismatching condition only under the further assumption that these features are relevant as movement triggers.

^14^We do not have space here to discuss the restriction that seems to force pronouns used as determiners to be either 1st or 2nd (and marginally 3rd) person plural in most languages. The interested reader should refer to Postal (1966), but also check the footnote in (Elbourne, 2005):60 reporting usage of first person singular in Early Modern English.

^15^*g* is a variable assignment, *a* is the addressee, ≤ _*i*_ the *individual part-of* relation Link (1983).

^16^The integration cost is calculated as crossing the verbal event (+1) and 2 or 3 nominal referents (+2/+3 respectively for *Art* and *Pro*). The cost of +2 for *Art* is considered for uniformity with respect the *top-down* model. These are the assumptions leading to the best possible predictions for the *memory-load* model. As for the *top-down* model, the FRC predicted is: Art_1_-Art_2_ = 9·3 = 27; Art_1_-Pro_2_ = 8·3 = 24; Pro_1_-Art_2_ = 9·3 = 27; Pro_1_-Pro_2_ = 16·3 = 48; See Supplementary Materials, section ACT-R-Based Model and section Top-Down Minimalist model for details.

^17^“≥” indicates a numerical, though non-significant difference; “>” indicates a significant difference.
