# Supplementary material for: Person Features and Lexical Restrictions in Italian Clefts
Source: Front Psychol. 2019 Sep 20;10:2105. doi: 10.3389/fpsyg.2019.02105 (PMC6764083; doi:10.3389/fpsyg.2019.02105)
Supplement: Supplementary file 1 [file Data_Sheet_1.PDF]

# *Supplementary Material*

# Person features and lexical restrictions in Italian clefts

**Cristiano Chesi*, Paolo Canal**

*** Correspondence:** Corresponding Author: cristiano.chesi@iusspavia.it

## 1 Experimental Items

| Condition | Item | Comprehension Question | Correct answer | Question topic | PP in question |
|---|---|---|---|---|---|
| $Art_1$-$Art_2$ | Sono i professori che i presidi hanno elogiato alla riunione d'istituto. | Qualcuno ha elogiato i professori alla riunione? | yes | obj | yes |
| $Art_1$-$Pro_2$ | Sono i professori che voi presidi avete elogiato alla riunione d'istituto. | I presidi hanno elogiato qualcuno alla riunione? | yes | subj | yes |
| $Pro_1$-$Art_2$ | Siete voi professori che i presidi hanno elogiato alla riunione d'istituto. | I professori hanno elogiato qualcuno alla riunione? | no | subj | yes |
| $Pro_1$-$Pro_2$ | Siete voi professori che voi presidi avete elogiato alla riunione d'istituto. | Qualcuno ha elogiato i presidi alla riunione? | no | obj | yes |
| $Art_1$-$Art_2$ | Sono gli avvocati che i giudici hanno evitato alla festa di inaugurazione. | Gli avvocati hanno evitato qualcuno alla festa? | no | obj | yes |
| $Art_1$-$Pro_2$ | Sono gli avvocati che voi giudici avete evitato alla festa di inaugurazione. | I giudici hanno evitato qualcuno alla festa? | yes | obj | yes |
| $Pro_1$-$Art_2$ | Siete voi avvocati che i giudici hanno evitato alla festa di inaugurazione. | Qualcuno ha evitato gli avvocati alla festa? | yes | subj | yes |
| $Pro_1$-$Pro_2$ | Siete voi avvocati che voi giudici avete evitato alla festa di inaugurazione. | Qualcuno ha evitato i giudici alla festa? | no | subj | yes |
| $Art_1$-$Art_2$ | Sono i politici che i sindacalisti hanno criticato alla manifestazione in piazza. | I sindacalisti hanno criticato qualcuno alla manifestazione? | no | subj | yes |
| $Art_1$-$Pro_2$ | Siete voi politici che voi sindacalisti avete criticato alla manifestazione in piazza. | Qualcuno ha criticato i sindacalisti alla manifestazione? | no | obj | yes |
| $Pro_1$-$Art_2$ | Sono i politici che i sindacalisti hanno criticato alla manifestazione in piazza. | Qualcuno ha criticato i politici alla manifestazione? | yes | obj | yes |
| $Pro_1$-$Pro_2$ | Siete voi politici che voi sindacalisti avete criticato alla manifestazione in piazza. | I politici hanno criticato qualcuno alla manifestazione? | yes | subj | yes |
| $Art_1$-$Art_2$ | Sono i turisti che i bagnini hanno aiutato sulla spiaggia affollata. | I bagnini hanno aiutato qualcuno sulla spiaggia? | yes | subj | yes |
| $Art_1$-$Pro_2$ | Sono i turisti che voi bagnini avete aiutato sulla spiaggia affollata. | I turisti hanno aiutato qualcuno sulla spiaggia? | no | subj | yes |
| $Pro_1$-$Art_2$ | Siete voi turisti che i bagnini hanno aiutato sulla spiaggia affollata. | Qualcuno ha aiutato i bagnini sulla spiaggia? | no | obj | yes |
| $Pro_1$-$Pro_2$ | Siete voi turisti che voi bagnini avete aiutato sulla spiaggia affollata. | Qualcuno ha aiutato i turisti sulla spiaggia? | yes | obj | yes |
| $Art_1$-$Art_2$ | Sono i banchieri che i funzionari hanno lodato davanti alla folla. | Qualcuno ha lodato i banchieri? | yes | obj | no |
| $Art_1$-$Pro_2$ | Sono i banchieri che voi funzionari avete lodano davanti alla folla. | I funzionari hanno lodato qualcuno? | yes | subj | no |
| $Pro_1$-$Art_2$ | Siete voi banchieri che i funzionari hanno lodato davanti alla folla. | I banchieri hanno lodato qualcuno? | no | subj | no |
| $Pro_1$-$Pro_2$ | Siete voi banchieri che voi funzionari avete lodano davanti alla folla. | Qualcuno ha lodato i funzionari? | no | obj | no |
| $Art_1$-$Art_2$ | Sono i ballerini che i coreografi hanno ringraziato alla prima dello spettacolo. | Qualcuno ha ringraziato i ballerini? | no | obj | no |
| $Art_1$-$Pro_2$ | Sono i ballerini che voi coreografi avete ringraziato alla prima dello spettacolo. | Qualcuno ha ringraziato i coreografi? | yes | obj | no |
| $Pro_1$-$Art_2$ | Siete voi ballerini che i coreografi hanno ringraziato alla prima dello spettacolo. | I coreografi hanno ringraziato qualcuno? | yes | subj | no |
| $Pro_1$-$Pro_2$ | Siete voi ballerini che voi coreografi avete ringraziato alla prima dello spettacolo. | I ballerini hanno ringraziato qualcuno? | no | subj | No |

| | | | | | |
|---|---|---|---|---|---|
| $Art_1$-$Art_2$ | Sono gli architetti che gli ingegneri hanno consultato prima di iniziare i lavori. | Gli architetti hanno consultato qualcuno? | no | subj | no |
| $Art_1$-$Pro_2$ | Sono gli architetti che voi ingegneri avete consultato prima di iniziare i lavori. | Qualcuno ha consultato gli ingegneri? | no | obj | no |
| $Pro_1$-$Art_2$ | Siete voi architetti che gli ingegneri hanno consultato prima di iniziare i lavori. | Qualcuno ha consultato gli architetti? | yes | obj | no |
| $Pro_1$-$Pro_2$ | Siete voi architetti che voi ingegneri avete consultato prima di iniziare i lavori. | Gli ingegneri hanno consultato qualcuno? | yes | subj | no |
| $Art_1$-$Art_2$ | Sono i ministri che i deputati hanno insultato durante la sessione parlamentare. | I deputati hanno insultato qualcuno? | yes | subj | no |
| $Art_1$-$Pro_2$ | Sono i ministri che voi deputati avete insultato durante la sessione parlamentare. | I ministri hanno insultato qualcuno? | no | subj | no |
| $Pro_1$-$Art_2$ | Siete voi ministri che i deputati hanno insultato durante la sessione parlamentare. | Qualcuno ha insultato i deputati? | no | obj | no |
| $Pro_1$-$Pro_2$ | Siete voi ministri che voi deputati avete insultato durante la sessione parlamentare. | Qualcuno ha insultato i ministri? | yes | obj | no |
| $Art_1$-$Art_2$ | Sono i custodi che i postini hanno interpellato per aprire la cassetta. | Qualcuno ha interpellato i custodi per aprire la cassetta? | yes | obj | yes |
| $Art_1$-$Pro_2$ | Sono i custodi che voi postini avete interpellato per aprire la cassetta. | I postini hanno interpellato qualcuno per aprire la cassetta? | yes | subj | yes |
| $Pro_1$-$Art_2$ | Siete voi custodi che i postini hanno interpellato per aprire la cassetta. | I custodi hanno interpellato qualcuno per aprire la cassetta? | no | subj | yes |
| $Pro_1$-$Pro_2$ | Siete voi custodi che voi postini avete interpellato per aprire la cassetta. | Qualcuno ha interpellato i postini per aprire la cassetta? | no | obj | yes |
| $Art_1$-$Art_2$ | Sono gli scrittori che gli editori hanno sollecitato alla fine della fiera. | Qualcuno ha sollecitato gli editori alla fine della fiera? | no | obj | yes |
| $Art_1$-$Pro_2$ | Sono gli scrittori che voi editori avete sollecitato alla fine della fiera. | Qualcuno ha sollecitato gli scrittori alla fine della fiera? | yes | obj | yes |
| $Pro_1$-$Art_2$ | Siete voi scrittori che gli editori hanno sollecitato alla fine della fiera. | Gli editori hanno sollecitato qualcuno alla fine della fiera? | yes | subj | yes |
| $Pro_1$-$Pro_2$ | Siete voi scrittori che voi editori avete sollecitato alla fine della fiera. | Gli scrittori hanno sollecitato qualcuno alla fine della fiera? | no | subj | yes |
| $Art_1$-$Art_2$ | Sono gli attori che i registi hanno incoraggiato prima dello spettacolo. | Gli attori hanno incoraggiato qualcuno prima dello spettacolo? | no | subj | yes |
| $Art_1$-$Pro_2$ | Sono gli attori che voi registi avete incoraggiato prima dello spettacolo. | Qualcuno ha incoraggiato i registi prima dello spettacolo? | no | obj | yes |
| $Pro_1$-$Art_2$ | Siete voi attori che i registi hanno incoraggiato prima dello spettacolo. | Qualcuno ha incoraggiato gli attori prima dello spettacolo? | yes | obj | yes |
| $Pro_1$-$Pro_2$ | Siete voi attori che voi registi avete incoraggiato prima dello spettacolo. | I registi hanno incoraggiato qualcuno prima dello spettacolo? | yes | subj | yes |
| $Art_1$-$Art_2$ | Sono i camerieri che i cassieri hanno rimproverato dopo la chiusura del ristorante. | I cassieri hanno rimproverato qualcuno dopo la chiusura? | yes | subj | yes |
| $Art_1$-$Pro_2$ | Sono i camerieri che voi cassieri avete rimproverato dopo la chiusura del ristorante. | I camerieri hanno rimproverato qualcuno dopo la chiusura? | no | subj | yes |
| $Pro_1$-$Art_2$ | Siete voi camerieri che i cassieri avete rimproverato dopo la chiusura del ristorante. | Qualcuno ha rimproverato i camerieri dopo la chiusura? | no | obj | yes |
| $Pro_1$-$Pro_2$ | Siete voi camerieri che voi cassieri avete rimproverato dopo la chiusura del ristorante. | Qualcuno ha rimproverato i camerieri dopo la chiusura? | yes | obj | yes |
| $Art_1$-$Art_2$ | Sono i violinisti che i pianisti hanno accompagnato durante tutto il concerto. | Qualcuno ha accompagnato i violinisti? | yes | obj | no |
| $Art_1$-$Pro_2$ | Sono i violinisti che voi pianisti avete accompagnato durante tutto il concerto. | I pianisti hanno accompagnato qualcuno? | yes | subj | no |
| $Pro_1$-$Art_2$ | Siete voi violinisti che i pianisti hanno accompagnato durante tutto il concerto. | I violinisti hanno accompagnato qualcuno? | no | subj | no |
| $Pro_1$-$Pro_2$ | Siete voi violinisti che voi pianisti avete accompagnato durante tutto il concerto. | Qualcuno ha accompagnato i pianisti? | no | obj | no |
| $Art_1$-$Art_2$ | Sono i tirocinanti che gli stagisti hanno interrogato dopo la visita in azienda. | Qualcuno ha interrogato gli stagisti? | no | obj | no |
| $Art_1$-$Pro_2$ | Sonogli istruttori che voi stagisti avete interrogato dopo la visita in azienda. | Qualcuno ha interrogato i tirocinanti? | yes | obj | no |
| $Pro_1$-$Art_2$ | Siete voi istruttori che gli stagisti hanno interrogato dopo la visita in azienda. | Gli stagisti hanno interrogato qualcuno? | yes | subj | no |
| $Pro_1$-$Pro_2$ | Siete voi istruttori che voi stagisti avete interrogato dopo la visita in azienda. | I tirocinanti hanno interrogato qualcuno? | no | subj | no |

| | | | | | |
|---|---|---|---|---|---|
| $Art_1$-$Art_2$ | Sono gli autori che i lettori hanno ascoltato dopo la presentazione dei libri. | Gli autori hanno ascoltato qualcuno? | no | subj | no |
| $Art_1$-$Pro_2$ | Sono gli autori che voi lettori avete ascoltato dopo la presentazione dei libri. | Qualcuno ha ascoltato i lettori? | no | obj | no |
| $Pro_1$-$Art_2$ | Siete voi autori che i lettori hanno ascoltato dopo la presentazione dei libri. | Qualcuno ha ascoltato gli autori? | yes | obj | no |
| $Pro_1$-$Pro_2$ | Siete voi autori che voi lettori avete ascoltato dopo la presentazione dei libri. | I lettori hanno ascoltato qualcuno? | yes | subj | no |
| $Art_1$-$Art_2$ | Sono i costumisti che i clienti hanno contattano al banco dei vestiti. | I clienti hanno contattato qualcuno? | yes | subj | no |
| $Art_1$-$Pro_2$ | Sono i costumisti che voi clienti avete contattato al banco dei vestiti. | I costumisti hanno contattato qualcuno? | no | subj | no |
| $Pro_1$-$Art_2$ | Siete voi costumisti che i clienti hanno contattano al banco dei vestiti. | Qualcuno ha contattato i clienti? | no | obj | no |
| $Pro_1$-$Pro_2$ | Siete voi costumisti che voi clienti avete contattato al banco dei vestiti. | Qualcuno ha contattato i costumisti? | yes | obj | no |
| $Art_1$-$Art_2$ | Sono i marinai che i passeggeri hanno avvertito prima del naufragio. | Qualcuno ha avvertito i marinai prima del naufragio? | yes | obj | yes |
| $Art_1$-$Pro_2$ | Sono i marinai che voi passeggeri hanno avvertito prima del naufragio. | I passeggeri hanno avvertito qualcuno prima del naufragio? | yes | subj | yes |
| $Pro_1$-$Art_2$ | Siete voi marinai che i passeggeri hanno avvertito prima del naufragio. | I marinai hanno avvertito qualcuno prima del naufragio? | no | subj | yes |
| $Pro_1$-$Pro_2$ | Siete voi marinai che voi passeggeri hanno avvertito prima del naufragio. | Qualcuno ha avvertito i passeggeri prima del naufragio? | no | obj | yes |
| $Art_1$-$Art_2$ | Sono i giocatori che gli allenatori hanno incolpato per l'esito della partita. | Qualcuno ha incolpato gli allenatori per l'esito della gara? | no | obj | yes |
| $Art_1$-$Pro_2$ | Sono i giocatori che voi allenatori avete incolpato per l'esito della partita. | Qualcuno ha incolpato i giocatori per l'esito della gara? | yes | obj | yes |
| $Pro_1$-$Art_2$ | Siete voi giocatori che gli allenatori hanno incolpato per l'esito della partita. | Gli allenatori hanno incolpato qualcuno per l'esito della gara? | yes | subj | yes |
| $Pro_1$-$Pro_2$ | Siete voi giocatori che voi allenatori avete incolpato per l'esito della partita. | I giocatori hanno incolpato qualcuno per l'esito della gara? | no | subj | yes |
| $Art_1$-$Art_2$ | Sono gli idraulici che gli elettricisti hanno visto nella casa in ristrutturazione. | Gli idraulici hanno visto qualcuno nella casa? | no | subj | yes |
| $Art_1$-$Pro_2$ | Sono gli idraulici che voi elettricisti avete visto nella casa in ristrutturazione. | Qualcuno ha visto gli elettricisti nella casa? | no | obj | yes |
| $Pro_1$-$Art_2$ | Siete voi idraulici che gli elettricisti hanno visto nella casa in ristrutturazione. | Qualcuno ha visto gli idraulici nella casa? | yes | obj | yes |
| $Pro_1$-$Pro_2$ | Siete voi idraulici che voi elettricisti avete visto nella casa in ristrutturazione. | Gli elettricisti hanno visto qualcuno nella casa? | yes | subj | yes |
| $Art_1$-$Art_2$ | Sono i critici che gli artisti hanno impressionato al concorso internazionale. | Gli artisti hanno impressionato qualcuno al concosrso? | yes | subj | yes |
| $Art_1$-$Pro_2$ | Sono i critici che voi artistiavete impressionato al concorso internazionale. | I critici hanno impressionato qualcuno al concosrso? | no | subj | yes |
| $Pro_1$-$Art_2$ | Siete voi critici che gli artisti hanno impressionato al concorso internazionale. | Qualcuno ha impressionato gli artisti al concosrso? | no | obj | yes |
| $Pro_1$-$Pro_2$ | Siete voi critici che voi artistiavete impressionato al concorso internazionale. | Qualcuno ha impressionato i giurati al concosrso? | yes | obj | yes |
| $Art_1$-$Art_2$ | Sono gli inquilini che i locatori hanno disapprovato durante la riunione di condominio. | Qualcuno ha disapprovato gli inquilini? | yes | obj | no |
| $Art_1$-$Pro_2$ | Sono gli inquilini che voi locatori avete disapprovato durante la riunione di condominio. | I locatori hanno disapprovato qualcuno? | yes | subj | no |
| $Pro_1$-$Art_2$ | Siete voi inquilini che i locatori hanno disapprovato durante la riunione di condominio. | Gli inquilini hanno disapprovato qualcuno? | no | subj | no |
| $Pro_1$-$Pro_2$ | Siete voi inquilini che voi locatori avete disapprovato durante la riunione di condominio. | Qualcuno ha disapprovato i locatori? | no | obj | no |
| $Art_1$-$Art_2$ | Sono i giornalisti che i consiglieri hanno accolto all'entrata del palazzo. | Qualcuno ha accolto i consiglieri? | no | obj | no |
| $Art_1$-$Pro_2$ | Sono i giornalisti che voi consiglieri avete accolto all'entrata del palazzo. | Qualcuno ha accolto i giornalisti | yes | obj | no |
| $Pro_1$-$Art_2$ | Siete voi giornalisti che voi consiglieri avete accolto all'entrata del palazzo. | I consiglieri hanno accolto qualcuno? | yes | subj | no |
| $Pro_1$-$Pro_2$ | Siete voi giornalisti che voi consiglieri avete accolto all'entrata del palazzo. | I consiglieri hanno accolto qualcuno? | no | subj | no |

| | | | | | |
|---|---|---|---|---|---|
| $Art_1$-$Art_2$ | Sono i pagliacci che i domatori hanno preceduto all'ingresso in sala. | I pagliacci hanno preceduto qualcuno? | no | subj | no |
| $Art_1$-$Pro_2$ | Sono i pagliacci che voi domatori avete preceduto all'ingresso in sala. | Qualcuno ha preceduto i domatori? | no | obj | no |
| $Pro_1$-$Art_2$ | Siete voi pagliacci che voi domatori avete preceduto all'ingresso in sala. | Qualcuno ha preceduto i pagliacci? | yes | obj | no |
| $Pro_1$-$Pro_2$ | Siete voi pagliacci che i domatori hanno preceduto all'ingresso in sala. | I domatori hanno preceduto qualcuno? | yes | subj | no |
| $Art_1$-$Art_2$ | Sono i giardinieri che i decoratori hanno supportato per la sistemazione del cortile. | Qualcuno ha supportato i giardinieri? | yes | subj | no |
| $Art_1$-$Pro_2$ | Sono i giardinieri che voi decoratori avete supportato per la sistemazione del cortile. | Qualcuno ha supportato i decoratori? | no | subj | no |
| $Pro_1$-$Art_2$ | Siete voi giardinieri che i decoratori hanno supportato per la sistemazione del cortile. | Qualcuno ha supportato i decoratori? | no | obj | no |
| $Pro_1$-$Pro_2$ | Siete voi giardinieri che voi decoratori avete supportato per la sistemazione del cortile. | Qualcuno ha supportato i giardinieri? | yes | obj | no |
| $Art_1$-$Art_2$ | Sono gli affittuari che i proprietari hanno denunciato alla guardia di finanza. | Qualcuno ha denunciato gli affittuari alla guardia di finanza? | yes | obj | yes |
| $Art_1$-$Pro_2$ | Sono gli affittuari che voi proprietari avete denunciato alla guardia di finanza. | Qualcuno ha denunciato i proprietari alla guardia di finanza? | yes | subj | yes |
| $Pro_1$-$Art_2$ | Siete voi affittuari che i proprietari hanno denunciato alla guardia di finanza. | Qualcuno ha denunciato i proprietari alla guardia di finanza? | no | subj | yes |
| $Pro_1$-$Pro_2$ | Siete voi affittuari che voi proprietari avete denunciato alla guardia di finanza. | Qualcuno ha denunciato gli affittuari alla guardia di finanza? | no | obj | yes |
| $Art_1$-$Art_2$ | Sono gli infermieri che i primari hanno chiamato in sala operatoria. | Qualcuno ha chiamato i primari in sala operatoria? | no | obj | yes |
| $Art_1$-$Pro_2$ | Sono gli infermieri che voi primari avete chiamato in sala operatoria. | Qualcuno ha chiamato gli infermieri in sala operatoria? | yes | obj | yes |
| $Pro_1$-$Art_2$ | Siete voi infermieri che i primari hanno chiamato in sala operatoria. | I primari hanno chiamato qualcuno in sala operatoria? | yes | subj | yes |
| $Pro_1$-$Pro_2$ | Siete voi infermieri che voi primari avete chiamato in sala operatoria. | Gli infermieri hanno chiamato qualcuno in sala operatoria? | no | subj | yes |
| $Art_1$-$Art_2$ | Sono i consulenti che gli amministratori hanno offeso nella sala conferenze. | Gli amministratori hanno lodato qualcuno nella sala conferenze? | no | subj | yes |
| $Art_1$-$Pro_2$ | Sono i consulenti che voi amministratori avete offeso nella sala conferenze. | Qualcuno ha incoraggiato i consulenti nella sala conferenze? | no | obj | yes |
| $Pro_1$-$Art_2$ | Siete voi consulenti che gli amministratori hanno offeso nella sala conferenze. | Qualcuno ha offeso i consulenti nella sala conferenze? | yes | obj | yes |
| $Pro_1$-$Pro_2$ | Siete voi consulenti che voi amministratori avete offeso nella sala conferenze. | Gli amministratori hanno offeso i consulenti nella sala conferenze? | yes | subj | yes |
| $Art_1$-$Art_2$ | Sono i tecnici che gli dirigenti hanno respinto dopo l'accesa discussione. | I dirigenti hanno respinto qualcuno dopo l'accesa discussione? | yes | subj | yes |
| $Art_1$-$Pro_2$ | Sono i tecnici che voi dirigenti avete respinto dopo l'accesa discussione. | I tecnici hanno respinto qualcuno dopo l'accesa discussione? | no | subj | yes |
| $Pro_1$-$Art_2$ | Siete voi tecnici che i dirigenti hanno respinto dopo l'accesa discussione. | Qualcuno ha respinto i dirigenti dopo l'accesa discussione? | no | obj | yes |
| $Pro_1$-$Pro_2$ | Siete voi tecnici che voi dirigenti avete respinto dopo l'accesa discussione. | Qualcuno ha respinto i tecnici dopo l'accesa discussione? | yes | obj | yes |
| $Art_1$-$Art_2$ | Sono i testimoni che gli imputati hanno screditato durante tutto il processo. | Qualcuno ha screditato i testimoni? | yes | obj | no |
| $Art_1$-$Pro_2$ | Sono i testimoni che voi imputati avete screditato durante tutto il processo. | Gli imputati hanno screditato qualcuno? | yes | subj | no |
| $Pro_1$-$Art_2$ | Siete voi testimoni che gli imputati hanno screditato durante tutto il processo. | I testimoni hanno screditato qualcuno? | no | subj | no |
| $Pro_1$-$Pro_2$ | Siete voi testimoni che voi imputati avete screditato durante tutto il processo. | Qualcuno ha screditato gli imputati? | no | obj | no |
| $Art_1$-$Art_2$ | Sono i geometri che i costruttori hanno accusato per il crollo del ponte. | Qualcuno ha accusato i costruttori? | no | obj | no |
| $Art_1$-$Pro_2$ | Sono i geometri che voi costruttori avete accusato per il crollo del ponte. | Qualcuno ha accusato i geometri? | yes | obj | no |
| $Pro_1$-$Art_2$ | Siete voi geometri che voi costruttori avete accusato per il crollo del ponte. | I costruttori hanno accusato qualcuno? | yes | subj | no |
| $Pro_1$-$Pro_2$ | Siete voi geometri che i costruttori hanno accusato per il crollo del ponte. | I geometri hanno accusato qualcuno? | no | subj | no |

| | | | | | |
|---|---|---|---|---|---|
| $Art_1$-$Art_2$ | Sono i macchinisti che i controllori hanno salutato alla stazione di Milano. | I macchinisti hanno salutato qualcuno? | no | subj | no |
| $Art_1$-$Pro_2$ | Sono i macchinisti che voi controllori avete salutato alla stazione di Milano. | Qualcuno ha salutato i controllori? | no | obj | no |
| $Pro_1$-$Art_2$ | Siete voi macchinisti che i controllori hanno salutato alla stazione di Milano. | Qualcuno ha salutato i macchinisti? | yes | obj | no |
| $Pro_1$-$Pro_2$ | Siete voi macchinisti che voi controllori avete salutato alla stazione di Milano. | I controllori hanno salutato qualcuno? | yes | subj | no |
| $Art_1$-$Art_2$ | Sono i cantanti che i musicisti hanno riconoscono al concerto di beneficenza. | I musicisti hanno riconosciuto qualcuno? | yes | subj | no |
| $Art_1$-$Pro_2$ | Sono i cantanti che voi musicisti avete riconosciuto al concerto di beneficenza. | I cantanti hanno salutato qualcuno? | no | subj | no |
| $Pro_1$-$Art_2$ | Siete voi cantanti che i musicisti hanno riconoscono al concerto di beneficenza. | Qualcuno ha riconosciuto i musicisti? | no | obj | no |
| $Pro_1$-$Pro_2$ | Siete voi cantanti che voi musicisti avete riconosciuto al concerto di beneficenza. | Qualcuno ha riconosciuto i cantanti? | yes | obj | no |

## 2 Specification of the formalisms and algorithmic procedures used

(1) Regions of Interest in the paradigm

a. $Art_1$-$Art_2$ (matching condition)
$|_{BE}$ **Sono** $|_{DP1}$ **gli** architetti $|_{C}$ che $|_{DP2}$ **gli** ingegneri $|_{VERB}$ **hanno** consultato $|_{SPILL}$ prima di iniziare i lavori|
*are$_{3P_PL}$* ***the*** *architects that* ***the*** *engineers* ***have$_{3P_PL}$*** *consulted before beginning the works*

b. $Art_1$-$Pro_2$ (mismatching condition)
$|_{BE}$ **Sono** $|_{DP1}$ **gli** architetti $|_{C}$ che $|_{DP2}$ **voi** ingegneri $|_{VERB}$ **avete** consultato $|_{SPILL}$ prima di iniziare i lavori|
*are$_{3P_PL}$* ***the*** *architects that* ***you*** *engineers* ***have$_{2P_PL}$*** *consulted before beginning the works*

c. $Pro_1$-$Art_2$ (mismatching condition)
$|_{BE}$ **Siete** $|_{DP1}$ **voi** architetti $|_{C}$ che $|_{DP2}$ **gli** ingegneri $|_{VERB}$ **hanno** consultato $|_{SPILL}$ prima di iniziare i lavori|
*are$_{2P_PL}$* ***you*** *architects that* ***the*** *engineers* ***have$_{3P_PL}$*** *consulted before beginning the works*

d. $Pro_1$-$Pro_2$ (matching condition)
$|_{BE}$ **Siete** $|_{DP1}$ **voi** architetti $|_{C}$ che $|_{DP2}$ **voi** ingegneri $|_{VERB}$ **avete** consultato $|_{SPILL}$ prima di iniziare i lavori|
*are$_{2P_PL}$* ***you*** *architects that* ***you*** *engineers* ***have$_{2P_PL}$*** *consulted before beginning the works*

### 2.1 Memory-load model

Below the numerical predictions associated to the Memory-load model following Gibson (1998, §2.2.2 main text). D. Grodner, E. Gibson (2005:266)

| | sono/ siete | gli/ voi | architetti | che | gli/ voi | ingegneri | hanno/ avete | consultato | prima | di | iniziare | i | lavori |
|---|---|---|---|---|---|---|---|---|---|---|---|---|---|
| New discourse referent | 1 | 0 | 1 | 0 | 0 | 1 | 0 | 1 | 1 | 0 | 1 | 0 | 1 |
| Structural integration | 0 | 0 | 0 | 0 | 0 | 0 | 0 | 2 | 0 | 0 | 0 | 0 | 0 |
| *Total* | *1* | *0* | *1* | *0* | *0* | *1* | *0* | *3* | *1* | *0* | *0* | *0* | *1* |

**Supplementary Table 1.** Word-by-word new discourse referent and structural integration cost (as in Gibson 1998).

| | | BE | DP1 | C | DP2 | VERB | SPILL |
|---|---|---|---|---|---|---|---|
| *$Art_1$-$Art_2$* | New discourse referent | 1 | 0.5 | 0 | 0.5 | 0.5 | 0.6 |
| | Structural integration | 0 | 0 | 0 | 0 | 1 | |
| | *Total* | *1* | *0.5* | *0* | *0.5* | *1.5* | *0.6* |
| *$Art_1$-$Pro_2$* | New discourse referent | 1 | 0.5 | 0 | 0.5 | 0.5 | 0.6 |
| | Structural integration | 0 | 0 | 0 | 0 | 1 | |
| | *Total* | *1* | *0.5* | *0* | *0.5* | *1.5* | *0.6* |
| *$Pro_1$-$Art_2$* | New discourse referent | 1 | 0.5 | 0 | 0.5 | 0.5 | 0.6 |
| | Structural integration | 0 | 0 | 0 | 0 | 1 | |
| | *Total* | *1* | *0.5* | *0* | *0.5* | *1.5* | *0.6* |
| *$Pro_1$-$Pro_2$* | New discourse referent | 1 | 0.5 | 0 | 0.5 | 0.5 | 0.6 |
| | Structural integration | 0 | 0 | 0 | 0 | 1 | |
| | *Total* | *1* | *0.5* | *0* | *0.5* | *1.5* | *0.6* |

**Supplementary Table 2.** Integration costs assuming *Pro* condition to have no discourse referent cost (as in Gibson 1998)

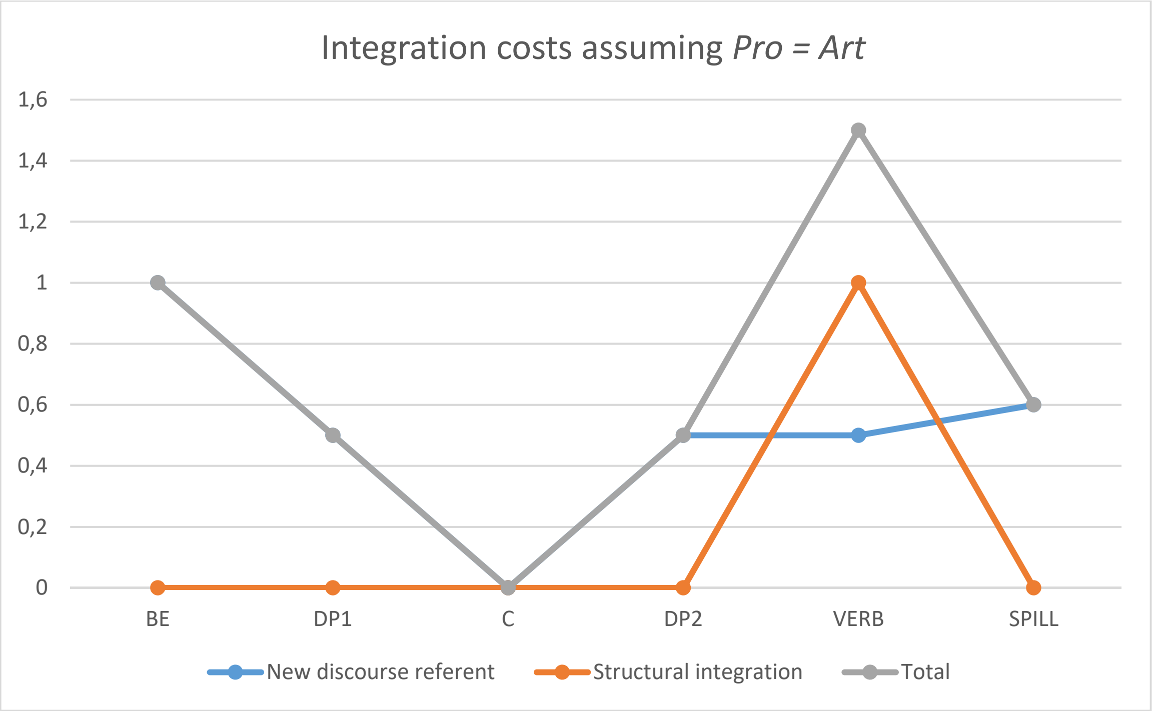


**Supplementary Figure 1.** Integration costs assuming *pro* having no discourse referent cost (data from **Supplementary Table 2**).

| *Conditions* | | ***BE*** | ***DP₁*** | ***C*** | ***DP₂*** | ***VERB*** | ***SPILL*** |
|---|---|---|---|---|---|---|---|
| *$Art_1$-$Art_2$* | New discourse referent | 1 | 2 | 1 | 2 | 1 | 4 |
| | Structural integration | 0 | 0 | 0 | 0 | 3 | 0 |
| | *Total* | *1* | 2 | 1 | 2 | *4* | 4 |
| *$Art_1$-$Pro_2$* | New discourse referent | 1 | 2 | 1 | 3 | 1 | 4 |
| | Structural integration | 0 | 0 | 0 | 0 | 4 | 0 |
| | *Total* | *1* | 2 | 1 | 3 | *5* | 4 |
| *$Pro_1$-$Art_2$* | New discourse referent | 1 | 3 | 1 | 2 | 1 | 4 |
| | Structural integration | 0 | 0 | 0 | 0 | 3 | 0 |
| | *Total* | *1* | 3 | 1 | 2 | *4* | 4 |
| *$Pro_1$-$Pro_2$* | New discourse referent | 1 | 3 | 1 | 3 | 1 | 4 |
| | Structural integration | 0 | 0 | 0 | 0 | 4 | 0 |
| | *Total* | *1* | 3 | 1 | 3 | *5* | 4 |

**Supplementary Table 3.** Integration costs assuming *Pro* condition in this context to have an extra +1 discourse referent cost, 1 cost unit both for C and BE, 2 cost units for the whole DP and 4 for SPILL.

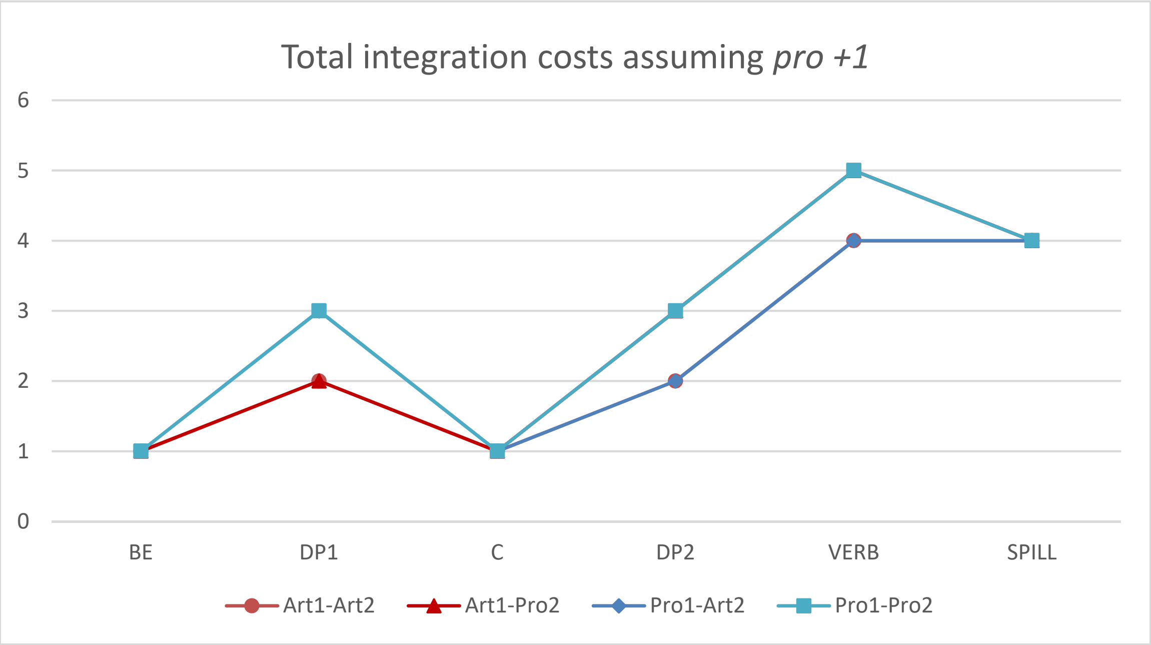


**Supplementary figure 2.** Total integration costs assuming *pro* contributing +1 as discourse referent costs (data from **Supplementary Table 3**).

## 2.2 ACT-R-based model

Lewis and Vasishth (2005) moment-by-moment model of parsing is based on the implementation of specific components of the Adaptive Control of Thought-Rational (ACT-R) architecture (Anderson et al 1997, Anderson 2005): two memory components are used in the ACT-R architecture for modeling retrieval in parsing, namely declarative memory and procedural memory. Declarative memory is used to store both lexical knowledge and chunks of the structure built on-line; this information is formally represented as Attribute-Value Matrices (as in HPSG, Pollard and Sag 1994, (2).b). Procedural memory consists of single-chunk buffers dealing with: (i) lexical access (ii) retrieved chunks from working memory and (iii) a control state (syntactic expectations expressed in terms of goals).

In order to operate, the procedure must postulate production rules, such as "*if* X *then* Y" (like in (2).c), based on linguistic knowledge expressed as standard rewriting rules (2).a (the example below is structurally simplified for sake of clarity, (2).d):

(2) a. IP → NP VP  DP → D NP  VP → V  NP→N
D → the  N → man  V → runs

b. IP $\begin{bmatrix} cat: & IP \\ num: & sing \\ spec: & DP \\ comp: & VP \\ tense: & pres \end{bmatrix}$ DP $\begin{bmatrix} cat: & DP \\ num: & sing \\ head: & the \\ comp: & NP \end{bmatrix}$

NP $\begin{bmatrix} cat: & NP \\ num: & sing \\ head: & man \end{bmatrix}$ VP $\begin{bmatrix} cat: & VP \\ num: & sing \\ person: & III \\ head: & runs \end{bmatrix}$

c. if DP is in spec-IP and AGREE[1], then set retrieve DP and attach it to IP

d.

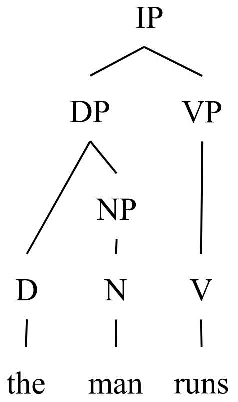


The model assumes a specific parsing algorithm compatible with the grammatical description used (Context-Free Grammars, Chomsky 1957): this is the Left-Corner (LC) algorithm (Aho & Ullmann

[1] AGREE can be defined conditionally as a boolean variable: set AGREE to TRUE if attached XP has features num and person with values underspecified or equals to num and person values of YP, otherwise set AGREE to FALSE.

1978); LC algorithm combines top-down expectations with bottom-up filtering strategy: assume we need to parse "the man runs" as in (2).d, given the knowledge expressed in (2).a and the syntactic chunks reported in (2).b, being "IP" the root node of a well-formed sentence, the parser should first expect "IP" at the beginning, then "the" is the first token to be integrated, hence it must be the "left corner" of the IP (namely the left-most lexical item to be rewritten below IP). If this is not the case, the sentence would be ungrammatical.

Given the toy-grammar in (2).a, we see that "the" is the left-most (unique, indeed) terminal rewritable item for D ("D → the"), which is the left-most item of NP ("NP → D N"), which is the left-most item of IP, hence "the" is a good left-corner for IP and can be attached. This is what the parser expected, hence [$_{IP}$ [$_{NP}$ [$_{D}$ the] …]] is the predicted partial structure to be updated in declarative memory, while N and VP (the remaining nodes resulted from the expansion of NP and S respectively) are pending goals to be associated to the next inputs in order for the structure to be grammatical.

From a procedural point of view:

1. when parsing starts, a word is attended and the relevant lexical item is accessed from declarative memory; this brings into the *lexical buffer* all information related to this lexical item (morphosyntactic, semantic features and argument structure);
2. the root goal is set at the beginning (according to the grammar in (2).b, "IP" is expected, then inserted in the *control state buffer*) and a production rule like "*if* X (in the lexical buffer) is a left corner of IP, *then* recursively replace the item in the *control state buffer* (a stack) with left-hand side of the rule" (i.e. "IP" will be replaced with "DP VP", then "DP VP" with "D N VP")
3. access working memory (this requires some milliseconds) and attach the pending item putting the constituent in the retrieval buffer;

SLASH features (Pollard and Sag 1994) are used to deal with non-local dependencies: whenever a VP with a gap/slash is processed, this must connect with the relevant argument already processed. The attachment time is simply proportional to the activation of the chunk to be retrieved as discussed in §2.3.4 (main article): activation of the chunks fades over time, then the longer is the time passed after the last activation, the longer it will take to retrieve it. The activation of a chunk is boosted whenever the chunk it is re-accessed.

No additional assumptions are made to tune this model with respect to the paradigm under study.

## 2.3 Top-Down Minimalist model

Given the lexicon in (3), the OC derivation for the relevant paradigm in (1)[2] is expanded step-by-step in (4):

(3) *Lex* = {[FocP =FinP], [FinP =S], [S =T], [T =V], [D =N], [N], [V], [(S) (FocP) D Dan$_i$ [N $t_i$]], [(S) (FocP) D John$_i$ [N $t_i$]], [(S) (FocP) D you [N Ø]], [(S) (FocP) D we [N Ø], [(S) (FocP) D the =N], [N banker], [N lawyer], [FinP that], [T V avoided =D [=D]]}

(4)

1. [FocP =FinP] (default root expectation)
2. [~~FocP~~ [~~FocP~~ D you [N Ø]] =FinP] (retrieve a compatible item from the lexicon (e.g. *you*) and merge it)
3. [~~FocP~~ [~~FocP~~ D you [N Ø]] =FinP] M=<[D /you/ [N Ø]]> (store unexpected features of *you* in memory (M))
4. [~~FocP~~ [~~FocP~~ D you [N Ø]] ~~=FinP~~[FinP =S]] (project a *FinP* expectation)
5. [~~FocP~~ [~~FocP~~ D you [N Ø]] ~~=FinP~~[~~FinP~~ that =S]] (retrieve a compatible lexical item from *Lex* (e.g. *that*) and merge it)
6. [~~FocP~~ [~~FocP~~ D you [N Ø]] ~~=FinP~~[~~FinP~~ that ~~=S~~[S =T]]] (project a *S* expectation)
7. [~~FocP~~ [~~FocP~~ D you [N Ø]] ~~=FinP~~[~~FinP~~ that ~~=S~~[S D Dan$_i$ [N $t_i$] =T]]] (retrieve a compatible lexical item from *Lex* (e.g. *Dan*) and merge it)
8. [~~FocP~~ [~~FocP~~ D you [N Ø]] ~~=FinP~~[~~FinP~~ that ~~=S~~[~~S~~ D Dan$_i$ [N $t_i$]] =T]] M=<[D /you/ [N Ø]], [D /Dan$_i$/ [N $t_i$]]> (store unexpected features of *Dan* in memory (M))
9. [~~FocP~~ [~~FocP~~ D you [N Ø]] ~~=FinP~~[~~FinP~~ that ~~=S~~[~~S~~ D Dan$_i$ [N $t_i$]] ~~=T~~ [T =V ]]] (project a *T* expectation)
10. [~~FocP~~ [~~FocP~~ D you [N Ø]] ~~=FinP~~[~~FinP~~ that ~~=S~~[~~S~~ D Dan$_i$ [N $t_i$]] ~~=T~~ [~~T~~ avoided ~~=V~~[~~V~~ /avoided/ =D [=D]]]]]] (retrieve a compatible lexical item from *Lex* (e.g. *avoided*) and merge it)
11. [~~FocP~~ [~~FocP~~ D you [N Ø]] ~~=FinP~~[~~FinP~~ that ~~=S~~[~~S~~ D Dan$_i$ [N $t_i$]] ~~=T~~ [~~T~~ avoided ~~=V~~[~~V~~ /avoided/ ~~=D~~[D =N] [=D]]]]] (project the first *D* (agent) expectation)
12. [~~FocP~~ [~~FocP~~ D you [N Ø]] ~~=FinP~~[~~FinP~~ that ~~=S~~[~~S~~ D Dan$_i$ [N $t_i$]] ~~=T~~ [~~T~~ avoided ~~=V~~[~~V~~ /avoided/ ~~=D~~[~~D~~ /Dan$_i$/ [N $t_i$]]] =D]]]] (retrieve and remove *Dan* from M, leaving M=<[D /you/ [N Ø]]>)
13. [~~FocP~~ [~~FocP~~ D you [N Ø]] ~~=FinP~~[~~FinP~~ that ~~=S~~[~~S~~ D Dan$_i$ [N $t_i$]] ~~=T~~ [~~T~~ avoided ~~=V~~[~~V~~ /avoided/ ~~=D~~[~~D~~ /Dan$_i$/ [N $t_i$]]] [~~=D~~ [D =N]]]]]] (project the last *D* (patient) expectation)
14. [~~FocP~~ [~~FocP~~ D you [N Ø]] ~~=FinP~~[~~FinP~~ that ~~=S~~[~~S~~ D Dan$_i$ [N $t_i$]] ~~=T~~ [~~T~~ avoided ~~=V~~[~~V~~ /avoided/ ~~=D~~[~~D~~ /Dan$_i$/ [N $t_i$]]] [~~=D~~ [~~D~~ /you/ [N Ø]]]]]]]] (retrieve and remove *you* from M, leaving M empty)

---

[2] *S* indicates the subject (topic-related) functional position (see Bianchi & Chesi 2014 for a discussion of the subject-related positions in this framework). Round brackets indicate optional features (S) and (FocP) can be associated to a *D* feature or not. Items between slashes have been already pronounced. The structure of the various DPs implements Longobardi's (1994-2005) analysis. Indexing à la Ealburne (2005), as well as anchoring of 1st and 2nd person will not be introduced for sake of simplicity but these are fully compatible with this featural specification (which can be represented as AVM, as in **Errore. L'origine riferimento non è stata trovata.**.b). Retrieval from *M*(emory) will always preempts lexical insertion; *M* is a last-in-first-out memory.

Given the Feature Retrieval Cost (*FRC*) in (5) and Feature Encoding Cost (*FEC*) in (6), *FREC* is calculated, phrase-by-phrase, as indicated in Supplementary Table 4.

(5) $$FRC(x) = \prod_{i=1}^{n} \frac{(1+nFi)^{m_i}}{(1+dFi)}$$

(6) $$FEC(x) = \sum_{i=1}^{n} eF_i$$

| *Conditions* | | ***BE*** | ***DP₁*** | ***C*** | ***DP₂*** | ***VERB*** | ***SPILL*** |
|---|---|---|---|---|---|---|---|
| *$Art_1$-$Art_2$* | FEC | 1 | 2 | 1 | 3 | 2 | 5 |
| | FRC | 0 | 0 | 0 | 0 | 1,43 | 0 |
| | *Total FREC* | 1 | 2 | 1 | 3 | 3,43 | 5 |
| *$Art_1$-$Pro_2$* | FEC | 1 | 2 | 1 | 4 | 2 | 5 |
| | FRC | 0 | 0 | 0 | 0 | 1,38 | 0 |
| | *Total FREC* | 1 | 2 | 1 | 4 | 3,38 | 5 |
| *$Pro_1$-$Art_2$* | FEC | 1 | 3 | 1 | 3 | 2 | 5 |
| | FRC | 0 | 0 | 0 | 0 | 1,43 | 0 |
| | *Total FREC* | 1 | 3 | 1 | 3 | 3,43 | 5 |
| *$Pro_1$-$Pro_2$* | FEC | 1 | 3 | 1 | 5 | 2 | 5 |
| | FRC | 0 | 0 | 0 | 0 | 1,68 | 0 |
| | *Total FREC* | 1 | 3 | 1 | 5 | 3,68 | 5 |

**Supplementary Table 4.** FRC+FEC (FREC)

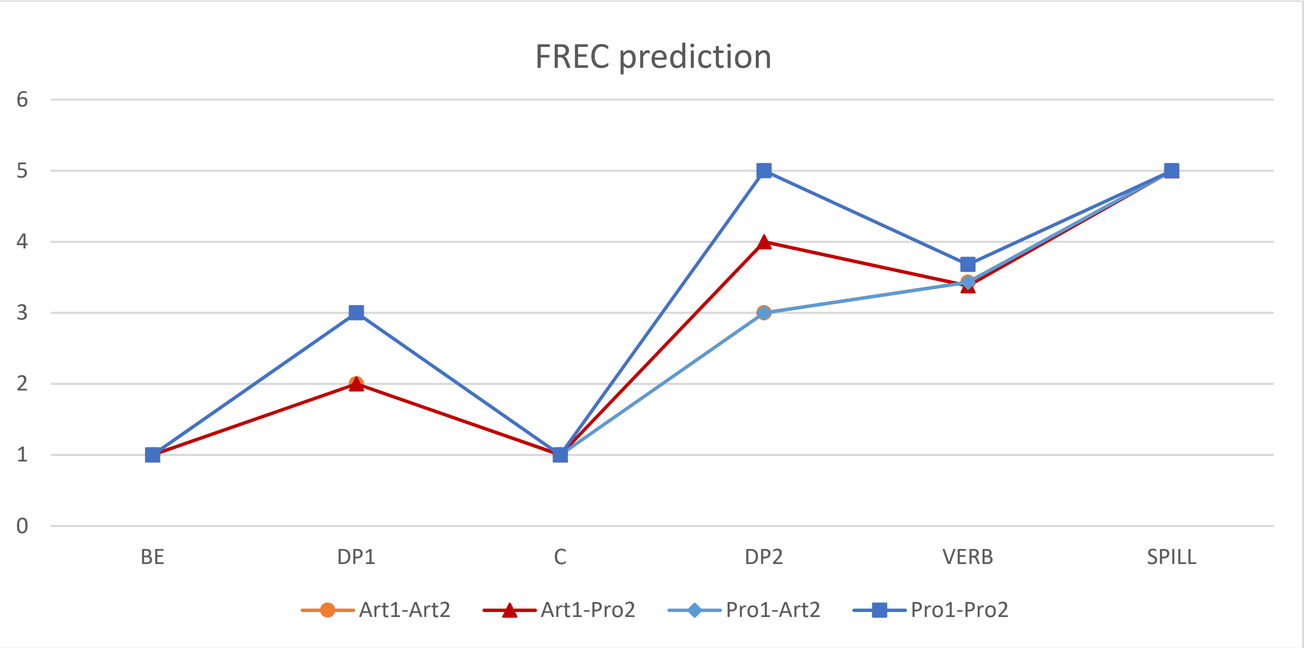


**Supplementary figure 3.** Total integration costs assuming *pro* contributing +1 as discourse referent costs (data from **Supplementary Table 4**).

In more details, in the paradigm in (1), FRC is only paid at the verbal predicate region (VERB) and it is calculated as follows, condition by condition:

$Art_1$-$Art_2$ = 9·3 = *27*; Log(27) = *1,43*.

Retrieving $Art_2$ costs 9, since *nF* = 2 (an index *D* and a nominal predicate *N*) + 1 (another distinct *N* is present in memory), *dF* = 0 (no distinct features are cued by the verb), *m* = 2 (two DPs are in memory).

$Art_1$ retrieval is paid 3, since *nF* = 2 (again, an index *D* and a nominal predicate *N*), *dF* = 0 and *m* = 0

$Art_1$-$Pro_2$ = 8·3 = *24*; Log(24) = *1,38*.

Retrieving $Pro_2$ costs 8, since *nF* = 2 (an index *D*, a nominal predicate *N*) + 1 (2nd person inclusion in the lexical restriction), *dF* = 1 (2nd person cued by the verb), *m* = 2 (two DPs are in memory).

$Art_1$ retrieval is paid 3, since *nF* = 2 (again, an index *D* and a nominal predicate *N*), *dF* = 0 and *m* = 0

$Pro_1$-$Art_2$ = 9·3 = 27; Log(27) = *1,43*.

Retrieving $Art_2$ costs 9, since *nF* = 2 (an index *D* and a nominal predicate *N*) + 1 (another distinct *N* is present in memory), *dF* = 0 (no distinct features are cued by the verb), *m* = 2 (two DPs are in memory).

$Pro_1$ retrieval is paid 3, since *nF* = 2 (again, an index *D* and a nominal predicate *N*) + 1 (2nd person inclusion in the lexical restriction), *dF* = 0 and *m* = 0

$Pro_1$-$Pro_2$ = 16·3 = 48; Log(48) = 1,68.

Retrieving $Pro_2$ costs 16, since *nF* = 2 (an index *D*, a nominal predicate *N*) + 1 (2nd person inclusion in the lexical restriction), *dF* = 0 (2nd person is cued by the verb, but two DPs in memory share this feature), *m* = 2 (two DPs are in memory).

$Pro_1$ retrieval is paid 3, since *nF* = 2 (again, an index *D* and a nominal predicate *N*) + 1 (2nd person inclusion in the lexical restriction), *dF* = 0 and *m* = 0

As for FEC (encoding cost) this is how features contribute, phrase by phrase, condition by condition (the phrases omitted in other conditions have a FEC cost identical with respect to the one indicated in the $Art_1$-$Art_2$ condition):

$Art_1$-$Art_2$

1. The *copula* introduces a tense specification. FEC = 1;
2. $Art_1$ introduces and index and a nominal predicate. FEC = 2;
3. *C* introduces a NP restriction. FEC = 1;
4. $Art_2$ introduces and index and a nominal predicate (FEC = 2), but this must be kept distinct from the one introduced by $Art_1$ since both are not yet attached to the relevant verbal predicate (FEC = +1). Total FEC = 3;
5. The *verbal predicate* introduces a tense and a predicate (FEC = 2);
6. The PP adjunct introduced a tense specification, a verbal predicate (FEC = 2) + a definite description (FEC = 2). Total FEC=4.

$Art_1$-$Pro_2$

2. *Art*$_1$ introduces and index and a nominal predicate. FEC = 2;
4. *Pro*$_2$ introduces and index and a nominal predicate (FEC = 2), but this must be kept distinct from the one introduced by *Art*$_1$ since both are not yet attached to the relevant verbal predicate (FEC = +1), moreover an encoding penalty for including the addressee in the NP predicate in this out of the blue context must be paid FEC = +1. Total FEC = 4;

*Pro*$_1$- *Art* $_2$

2. *Pro*$_1$ introduces and index, a nominal predicate (FEC = 2) and an encoding penalty for including the addressee in the NP predicate out of the blue (FEC = +1). Total FEC = 3;
4. *Art*$_2$ introduces and index and a nominal predicate (FEC = 2), but this must be kept distinct from the one introduced by *Pro*$_1$ since both are not yet attached to the relevant verbal predicate (FEC = +1). Total FEC = 3;

*Pro*$_1$- *Pro*$_2$

2. *Pro*$_1$ introduces and index, a nominal predicate (FEC = 2) and an encoding penalty for including the addressee in the NP predicate out of the blue (FEC = +1). Total FEC = 3;
4. *Pro*$_2$ introduces and index and a nominal predicate (FEC = 2), but this must be kept distinct from the one introduced by *Pro*$_1$ since both are not yet attached to the relevant verbal predicate (FEC = +1), moreover an encoding penalty for including the addressee in the NP predicate in this out of the blue context must be paid FEC = +1, with an additional cost (FEC = +1) due to the necessity of keeping the addressee distinct from the one introduced in *Pro*$_1$. Total FEC = 5;

An on-line tool, where these assumptions are encoded, can be directly used:
http://www.ciscl.unisi.it/top-down/frec.htm

The assumptions on the relevant features encoded and retrieved can be freely modified, to test additional assumptions and alternative hypotheses.
